# Developing an individualized risk calculator for psychopathology among young people victimized during childhood: A population-representative cohort study

**DOI:** 10.1016/j.jad.2019.10.034

**Published:** 2020-02-01

**Authors:** Alan J. Meehan, Rachel M. Latham, Louise Arseneault, Daniel Stahl, Helen L. Fisher, Andrea Danese

**Affiliations:** a Social, Genetic & Developmental Psychiatry Centre, Institute of Psychiatry, Psychology & Neuroscience, King's College London, London, UK; b Department of Biostatistics & Health Informatics, Institute of Psychiatry, Psychology & Neuroscience, King's College London, London, UK; c Department of Child & Adolescent Psychiatry, Institute of Psychiatry, Psychology & Neuroscience, King's College London, London, UK; d National and Specialist CAMHS Trauma, Anxiety, and Depression Clinic, South London and Maudsley NHS Foundation Trust, London, UK

**Keywords:** Victimization, Psychopathology, Risk prediction, Risk calculator, Resilience

## Abstract

**Background:**

Victimized children are at greater risk for psychopathology than non-victimized peers. However, not all victimized children develop psychiatric disorders, and accurately identifying which victimized children are at greatest risk for psychopathology is important to provide targeted interventions. This study sought to develop and internally validate individualized risk prediction models for psychopathology among victimized children.

**Methods:**

Participants were members of the Environmental Risk (E-Risk) Longitudinal Twin Study, a nationally-representative British birth cohort of 2,232 twins born in 1994–1995. Victimization exposure was measured prospectively between ages 5 and 12 years, alongside a comprehensive range of individual-, family-, and community-level predictors of psychopathology. Structured psychiatric interviews took place at age-18 assessment. Logistic regression models were estimated with Least Absolute Shrinkage and Selection Operator (LASSO) regularization to avoid over-fitting to the current sample, and internally validated using 10-fold nested cross-validation.

**Results:**

26.5% (*n* = 591) of E-Risk participants had been exposed to at least one form of severe childhood victimization, and 60.4% (*n* = 334) of victimized children met diagnostic criteria for any psychiatric disorder at age 18. Separate prediction models for any psychiatric disorder, internalizing disorders, and externalizing disorders selected parsimonious subsets of predictors. The three internally validated models showed adequate discrimination, based on area-under-the-curve estimates (range = =0.66–0.73), and good calibration.

**Limitations:**

External validation in wholly-independent data is needed before clinical implementation.

**Conclusions:**

Findings offer proof-of-principle evidence that prediction modeling can be useful in supporting identification of victimized children at greatest risk for psychopathology. This has the potential to inform targeted interventions and rational resource allocation.

## Highlights

• Three risk calculators were tested to predict psychopathology in victimized children.
• These prediction models quantify each child's unique risk for later psychopathology.
• All three risk calculators showed adequate discrimination and good calibration.
• This analytic approach could enhance clinical decision-making for victimized youth.

## 1 Introduction

Childhood victimization is associated with a range of internalizing (Nanni et al., 2012; Takizawa et al., 2014), externalizing (Braga et al., 2017; Capusan et al., 2016), and psychotic disorders (Fisher et al., 2013), likely reflecting a general vulnerability for psychopathology in victimized children (Schaefer et al., 2018). Yet, not all victimized children develop psychopathology (Rutter, 2013). Accurately identifying which victimized children are at greatest risk for psychopathology is therefore important, in order to provide targeted support and to inform rational allocation of resources. Previous studies have described individual-, family-, and community-level factors that increase or decrease risk of psychopathology among victimized children (i.e., vulnerability or resilience factors, respectively), and thus, may improve risk detection (Fritz et al., 2018; Meng et al., 2018). However, these studies also typically had important limitations: they were based on cross-sectional or short-term longitudinal designs, making it difficult to ascertain whether victimization and potential resilience/vulnerability factors preceded the onset of psychopathology (Cicchetti, 2013; Meng et al., 2018); they described resilience/vulnerability factors in isolation, likely over-estimating their individual contribution compared to more realistic multivariate models (Fritz et al., 2018); and they identified factors that modify the *average* risk for psychopathology for subgroups of victimized children with shared features, but did not test if such factors can accurately predict which victimized children do or do not develop psychopathology (Yarkoni and Westfall, 2017). As such, the extent to which established resilience/vulnerability factors can inform *individualized* risk prediction is unclear.

To address these gaps, we investigated psychiatric risk prediction among members of a nationally-representative prospective British cohort. Building on prediction modeling methods developed for medical conditions (Damen et al., 2016; Hippisley-Cox and Coupland, 2015) and adult psychiatric disorders (Cannon et al., 2016; Fusar-Poli et al., 2017; Hafeman et al., 2017), we developed multivariate individualized risk prediction models (risk calculators) for psychopathology among victimized children (Moons et al., 2009; Steyerberg and Vergouwe, 2014). We also internally validated our findings using a nested cross-validation approach to determine the classification accuracy of prediction models when applied to independent cases in our sample (Steyerberg, 2009).

## 2 Methods

### 2.1 Participants

Participants were members of the Environmental Risk (E-Risk) Longitudinal Twin Study, which tracks the development of a nationally-representative birth cohort of 2232 twin children born in England and Wales in 1994–1995. Full details about the sample are reported elsewhere (Moffitt and E-Risk Study Team, 2002) and in Supplementary Material. Briefly, the E-Risk sample was constructed in 1999–2000 when 1116 families (93% of those eligible) with same-sex 5-year-old twins participated in home-visit assessments. This sample comprised 56% monozygotic and 44% dizygotic twin pairs; sex was evenly distributed within zygosity (49% male). Families were recruited to represent the UK population of families with newborns in the 1990s, on the basis of residential location throughout England and Wales and mother's age.

Follow-up home-visits were conducted when children were aged 7, 10, 12, and 18 (participation rates were 98%, 96%, 96%, and 93%, respectively). At age 18, 2066 participants were assessed. Average age at time of assessment was 18.4 years (*SD* = 0.36); all interviews were conducted after the 18th birthday. There were no differences between those who did and did not take part at age 18 in terms of socioeconomic status, assessed when the cohort was initially defined (*χ*^2^ = 0.86, *p* = .65), age-5 IQ (*t *= 0.98, *p* = .33), age-5 behavioral (*t *= 0.40, *p* = .69) or emotional (*t *= 0.41, *p* = .68) problems, or childhood poly-victimization (*z *= 0.51, *p* = .61). The Joint South London and Maudsley and Institute of Psychiatry Research Ethics Committee approved each study phase. Parents gave informed consent, and twins gave assent between 5 and 12 years and then informed consent at age 18.

The present study focuses on a subset of the E-Risk Study sample who were exposed to any type of severe victimization during childhood (*n *= 591; 26.5% of sample; 50% male). Information about the victimization measure used to derive this analytic sample is provided below and in Supplementary Material. Based on multivariate logistic regression and odds ratios (ORs), victimized and non-victimized participants did not differ on the distribution of sex (OR = 0.92, 95% CI = 0.76–1.12, *p* = .40) and ethnicity (White vs non-White; OR = 1.00, 95% CI = 0.72–1.39, *p* = .99); however, victimized young people were more likely to be in the lowest social class tertile compared to their non-victimized counterparts (OR = 2.91, 95% CI = 2.40–3.54, *p* < 0.001).

### 2.2 Measures

#### 2.2.1 Childhood victimization

Prospective measures of victimization utilized in this cohort are described elsewhere (Danese et al., 2017; Fisher et al., 2015), and in Supplementary Material. In brief, lifetime exposure to several types of victimization was assessed repeatedly when children were 5, 7, 10, and 12 years of age. Comprehensive dossiers were compiled for each child with cumulative information about: exposure to domestic violence between mother and partner; frequent bullying by peers; physical abuse by an adult; sexual abuse; emotional abuse and neglect; and physical neglect, all between birth and age 12 years. Dossiers comprised reports from caregivers, recorded narratives of caregiver interviews, recorded debriefings with research workers who had coded any indications of abuse and neglect at any of the successive home visits, and information from clinicians whenever the study team made a child-protection referral. These were reviewed by two independent researchers and rated for the presence and severity (none/mild/severe) of each type of victimization. In the present study, prospectively-measured victimization was dichotomized to represent ‘none/mild’ (0) vs ‘severe’ (1) victimization.

#### 2.2.2 Age-18 psychopathology

Past-year psychopathological symptoms were comprehensively assessed through private interviews available at age-18 follow-up. Ten disorder diagnoses were organized into three domains (internalizing, externalizing, and thought disorders), based on a reliable latent factor structure for psychopathology previously identified within the E-Risk Study (Schaefer et al., 2018). Full information on individual diagnoses is available in Supplementary Material. In brief, in order to effectively evaluate the classification accuracy of each prediction model, binary classifications were derived for each domain, denoting the presence of at least one of the constituent disorders based on diagnostic cut-offs. Participants were classified as having ‘internalizing disorder’ if they met diagnostic criteria for generalized anxiety disorder, major depressive disorder or post-traumatic stress disorder, or presented at least 2 of 5 eating disorder symptoms from an established screening tool, indicating a possible case of anorexia nervosa or bulimia nervosa (Morgan et al., 1999). Participants were classified as having ‘externalizing disorder’ where they met diagnostic criteria for attention-deficit/hyperactivity disorder, conduct disorder, alcohol dependence, cannabis dependence, or tobacco dependence. Finally, ‘thought disorder’ classification was based on the definite presence of at least one of seven psychotic symptoms, centered on delusions and hallucinations. The measure has good construct validity, as it was shown to share many of genetic, social, neurodevelopmental, and behavioral risk factors and correlates as adult schizophrenia (Polanczyk et al., 2010). From these three domain-specific classifications, an overall binary outcome for ‘any psychiatric disorder’ was created, denoting the presence of any internalizing, externalizing, or thought disorder (1), or the absence of all three (0).

The prevalence of each disorder and wider diagnostic domain, within both the overall E-Risk sample and our victimized sub-sample, is presented in Table 1. Overall, out of 553 victimized participants, 60.4% met diagnostic criteria for any of the ten psychiatric disorders, 37.9% met criteria for internalizing disorder, and 43.3% met criteria for externalizing disorder. Regarding comorbidity between internalizing and externalizing domains, among those diagnosed with at least one of their nine underlying disorders, 36.4% (*n *= 118) had both an internalizing and externalizing disorder, 27.2% (*n *= 88) had an internalizing disorder alone, and 36.4% (*n *= 118) had an externalizing disorder alone.

**Table 1:** Prevalence of psychopathology at age 18 among (i) the main E-Risk sample, and (ii) a subsample of E-Risk participants exposed to severe childhood victimization.

	Main Sample (*N *= 2050–2066)		Victimized Sample (*N *= 551–558)	
Diagnosis	*N*	%	*N*	%
**Any psychiatric disorder**	973	47.5	334	60.4
**Internalizing disorder**	585	28.5	209	37.9
Generalized anxiety disorder	153	7.4	63	11.3
Major depressive disorder	414	20.1	163	29.2
Post-traumatic stress disorder	90	4.4	41	7.4
Eating disorder	204	9.9	71	12.9
**Externalizing disorder**	656	31.9	239	43.3
Attention-deficit/hyperactivity disorder	171	8.3	64	11.5
Conduct disorder	309	15.1	119	21.5
Alcohol dependence	263	12.8	90	16.1
Cannabis dependence	89	4.3	35	6.3
Tobacco dependence	183	8.9	85	15.3
**Thought disorder**	59	2.9	29	5.2

#### 2.2.3 Childhood predictors

Individual-, family-, and community-level predictors were assessed between ages 5 and 12 years. We utilized a recent systematic review of multi-level predictors of maltreatment outcomes (Meng et al., 2018) and mapped these predictors to variables measured in the E-Risk Study (see Supplementary **Tables S1** and **S2**). This approach is preferable to selecting predictors based on statistically-significant bivariate associations with the outcome in the target sample, as it avoids the circular logic of testing the predictive ability of variables already known to be associated with the outcome within that sample (Fusar-Poli et al., 2018). We identified 22 predictors, summarized in Table 2, with detailed descriptions in the Supplementary Material. With 22 predictors, we achieved a minimum events per variable (EPV) ratio of 10 for our main ‘any psychiatric disorder’ outcome, mitigating potential model instability due to over-fitting (Pavlou et al., 2015).

**Table 2:** Summary of childhood predictors.

Measure	Age	Informant	Description
***Individual***			
Sex	5	Mother	1 = Male; 2 = Female
IQ	12	Child	Pro-rated WISC-R score (Matrix Reasoning and Information subtests)
Openness to experience	12	Researcher	Sum of 5 BFI items
Conscientiousness			Sum of 6 BFI items
Extraversion			Sum of 6 BFI items
Agreeableness			Sum of 5 BFI items
Neuroticism			Sum of 5 BFI items
ADHD symptoms	12	Mother/teacher	18 DSM-IV inattentive and impulsive-hyperactive symptoms from CBCL (mothers) and TRF items (teachers), averaged across raters
Conduct disorder symptoms	12	Mother/teacher	14 DSM-IV criteria from CBCL (mothers) and TRF items (teachers), averaged across raters
Anxiety	12	Child	Symptom score from 10 MASC items
Depression	12	Child	Symptom score from 27 CDI items
Self-harm/suicide attempt	12	Mother	Any deliberate self-harm or attempted suicide in previous six months
Psychotic symptoms	12	Child	Presence of at least one definite psychotic symptom
***Family***			
Maternal warmth	5; 10	Mother	Warmth, enthusiasm, interest in, enjoyment of child during FMSS, summed across time-points
Sibling warmth	7; 10	Mother	6 items each, summed across time-points
Adult involvement	12	Child	13 items assessing presence of supportive adult
Family history of psychopathology	12	Mother	Proportion (0.0–1.0) of family members (parents, grandparents, aunts/uncles) with history of psychiatric disorder
Biological parents in household	10	Mother	Number of biological parents in household up to age 10 (0 = both always absent; 1 = one absent at some point; 2 = both always present)
Socioeconomic status	5	Mother	Tertiles derived from standardized composite of parental income, education, and occupation
***Community***			
Neighborhood crime victimization	5	Mother	3 items assessing family's experience of violent crime, burglary, or theft in local area
Social cohesion	5	Mother	Sum of 5 items (neighbors close-knit, share values, trust each other, etc.)
Status among peers	12	Child	Self-selected position within 5 peer status ‘circles’

*Notes.* For full details of measures with accompanying references, see Supplementary Material. Child's age given in years. ADHD = attention-deficit/hyperactivity disorder; BFI = Big Five Inventory; CBCL = Child Behavior Checklist; CDI = Children's Depression Inventory; DSM-IV = Diagnostic and Statistical Manual of Mental Disorders, Fourth Edition; FMSS = Five-Minute Speech Sample; MASC = Multidimensional Anxiety Scale for Children; TRF = Teacher's Report Form; WISC–R = Wechsler Intelligence Scale for Children–Revised.

#### 2.3 Statistical analyses

Analyses were conducted using STATA version 15.0 and R version 3.4.2. First, we tested whether the prevalence of each form of psychopathology significantly differed between victimized and non-victimized participants using logistic regression, correcting for familial clustering using the *cluster* STATA command.

Second, we developed and internally validated separate prediction models for three age-18 psychiatric outcomes using regularized logistic regression in the *glmnet* R package (Friedman et al., 2010). Specifically, we evaluated predictive ability for the overall measure of ‘any psychiatric disorder’, as well as separately for internalizing and externalizing disorders, as the two dimensions do show distinct features and risk profiles, even over and above a general factor for psychopathology (Lahey et al., 2017). We could not reliably estimate a separate model for thought disorder given the low prevalence of psychotic symptoms. Complete data were available in 91.3%, 91.5% and 91.3% of victimized children with available data for ‘any psychiatric’, ‘externalizing’ and ‘internalizing’ disorders, respectively (*n *= 504–505). Therefore, a complete-cases approach was utilized for model development and internal validation.

A full description of our analyses is available in Supplementary Material. Briefly, we conducted regularized regression using the Least Absolute Shrinkage and Selection Operator (LASSO) to identify subsets of predictors that maximized prediction accuracy for each outcome in unseen cases within the sample. LASSO shrinks coefficients towards zero, thereby reducing the variance of these estimates. Predictors whose coefficients are shrunk to zero are excluded from the model, enabling parsimonious solutions. The degree of shrinkage is determined by a tuning parameter, lambda (*λ*). Cross-validation was used to identify the optimal *λ* for a model (Tibshirani, 1996). To obtain an estimate of prediction accuracy in new cases from the same underlying population, we internally validated each model using nested 10-fold cross-validation (see Supplementary **Fig. S2**; Hastie et al., 2009).

Performance measures were based on each child's predicted probability when held out from model selection and estimation as an unseen ‘test case’. Discrimination (the model's ability to accurately classify those with and without psychopathology) was visualized using the receiver operator characteristic (ROC) curve, and quantified using the area-under-the-curve (AUC; Steyerberg, 2009). AUC ranges from 0.5 (chance-level) to 1 (perfect discrimination), with the following proposed benchmarks: <0.7 (poor); 0.7–0.8 (acceptable); 0.8–0.9 (excellent); 0.9–1.0 (outstanding; Hosmer et al., 2013). Calibration was assessed by plotting predicted probabilities (grouped into equal-interval bins) against observed outcomes, where a 45° line indicates perfect agreement. A chi-square test of unreliability (*U*) determined whether each calibration plot's intercept (‘calibration-in-the-large’) and slope significantly differed from the ideal line (Fenlon et al., 2018). Overall performance was assessed by the proportion of explained deviance (‘pseudo-*R*^2^’), and the Brier score, or mean squared difference between predicted probabilities and actual binary outcomes (Brier, 1950). We scaled Brier scores by their maximum possible values, which vary based on the incidence of the outcome, from 0% (non-informative) to 100% (perfect; Steyerberg, 2009)

Finally, we carried out two sensitivity analyses. First, we tested whether model performance and/or interpretability would improve if we allowed inclusion of a set of correlated data in our model. To do this, we re-estimated each model using elastic net regularization. Unlike LASSO, this regularization method selects or excludes sets of correlated variables, potentially allowing more predictors to be retained. Second, to test whether predictive ability was inflated by the presence of non-independent twin observations, we re-ran analyses in ten subsamples, each consisting of one twin per pair.

## 3 Results

### 3.1 Descriptive statistics

Victimized children were significantly more likely than their non-victimized peers to present with any psychiatric (OR = 2.05, 95% CI = 1.63–2.57, *p* < .001), internalizing (OR = 1.82, 95% CI = 1.44–2.30, *p* < .001), externalizing (OR = 1.99, 95% CI = 1.58–2.49, *p* < .001), or thought disorder at age 18 (OR = 2.70, 95% CI = 1.57–4.62, *p* < .001). However, a substantial proportion of victimized children did not have any psychiatric (39.6%), internalizing (62.1%), externalizing (56.7%), or thought (94.8%) disorder at age 18 (see Supplementary **Fig. S1**). Therefore, we developed prediction models to accurately identify individualized risk for psychopathology among victimized children.

### 3.2 Prediction modeling

Unstandardized coefficients for the predictors retained in each of the three models following LASSO variable selection (see Table 3) were obtained by re-estimating the cross-validated model using the average optimal *λ* tuning parameter, identified from ten repeated model development loops. Using each set of coefficients, we derived regression equations to estimate a child's individual risk for an outcome based on his/her values on these retained predictors (see Supplementary **Table S3**). Additionally, the classification accuracy achieved by each model across various thresholds of predicted risk are presented in Supplementary **Tables S4–S6**. Model composition and performance are described below for each outcome.

**Table 3:** Unstandardized coefficients for prediction models.

	**A**	**B**	**C**
	Any psychiatric disorder	Internalizing disorder	Externalizing disorder
	(*n *= 505)	(*n *= 504)	(*n *= 505)
Predictor	*B*	*B*	*B*
Intercept	2.186	−1.643	2.558
***Individual***			
Sex (female)	−0.291	.329	−0.811
IQ	−0.001	–	−0.008
Openness to experience	.038	.037	–
Conscientiousness	−0.018	−0.018	–
Extraversion	.107	.090	.048
Agreeableness	−0.166	−0.059	–
Neuroticism	–	.088	–
ADHD symptoms	–	–	.005
CD symptoms	.250	.063	.267
Anxiety symptoms	.029	.057	–
Depression symptoms	.002	.005	.005
Self-harm/suicide attempt	–	−0.416	–
Psychotic symptoms	1.044	1.112	.551
***Family***			
Maternal warmth	.033	.101	–
Sibling warmth	−0.027	–	−0.051
Adult involvement	−0.011	−0.031	−0.019
Family history of psychopathology	.323	.709	.038
Biological parents in household	−0.310	−0.106	−0.317
Socioeconomic status	−0.164	−0.194	−0.004
***Community***			
Neighborhood crime victimization	–	−0.060	.070
Social cohesion	–	–	−0.023
Status among peers	−0.047	−0.091	.067
			
Deviance explained (Pseudo-*R*^2^)	13.5%	11.1%	15.4%

*Notes.* For sex, positive values imply a greater number of females with that outcome, while negative values suggest greater prevalence among males. Regression equations for individual risk calculation, derived from these coefficients, are presented in Supplementary **Table S3**. B = unstandardized regression coefficient; ADHD = attention-deficit/hyperactivity disorder; CD = conduct disorder.

#### 3.2.1 Any psychiatric disorder

**Fig. 1:**
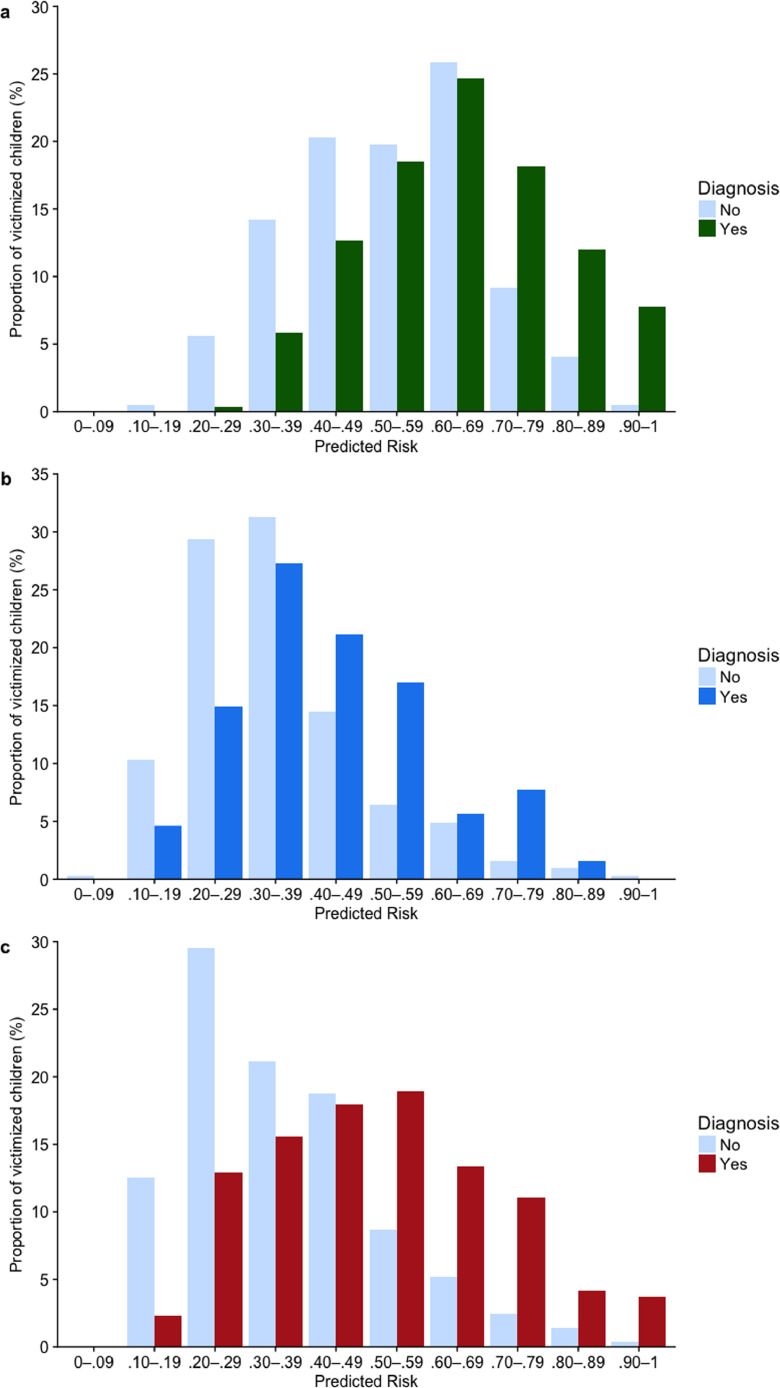
Frequency distributions of predicted risk among victimized children with and without (**a**) any psychiatric; (**b**) internalizing; and (**c**) externalizing disorder.

**Fig. 2:**
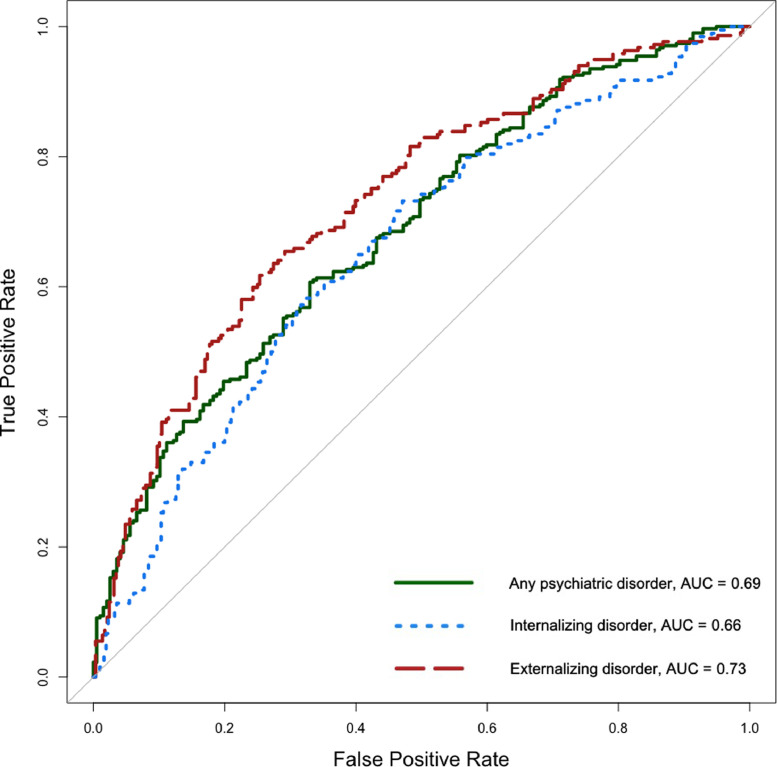
Receiver operating characteristic (ROC) curves for the three prediction models among victimized children. ROC curves plot the true positive rate (sensitivity; the proportion of actual positive outcomes correctly identified as such) against the false positive rate (1–specificity; the proportion of incorrectly-classified positive outcomes). The solid diagonal line denotes chance-level discrimination.

**Fig. 3:**
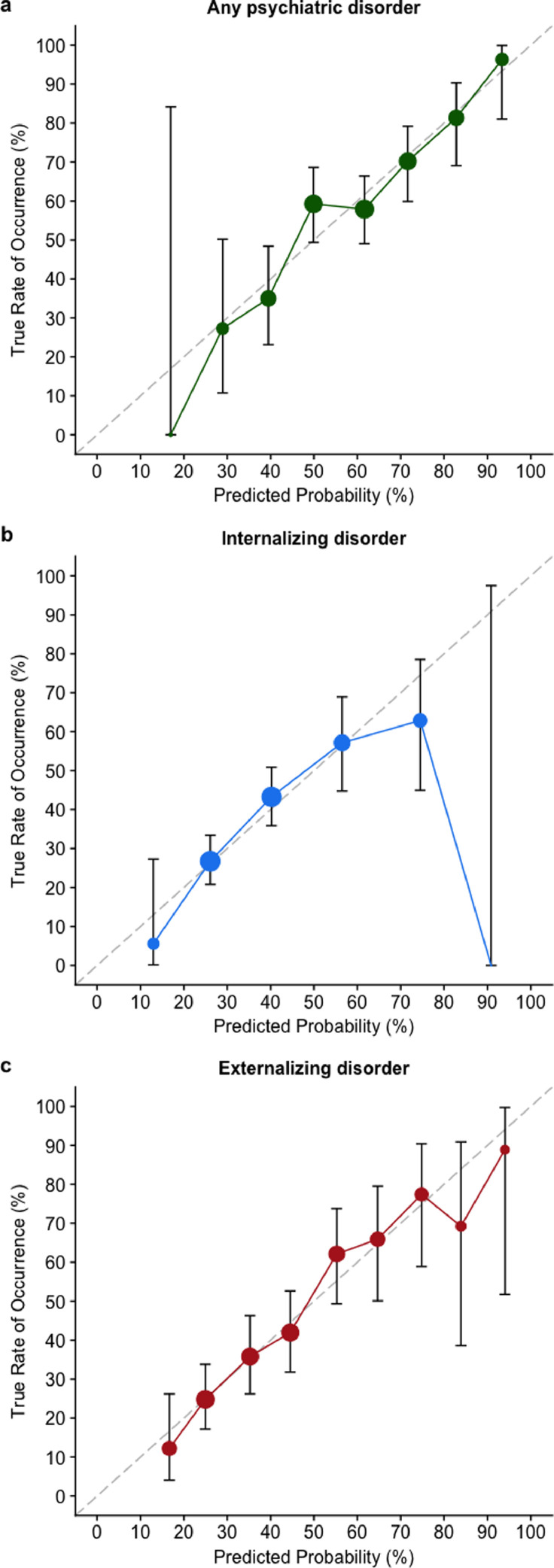
Calibration plots for (**a**) any psychiatric, (**b**) internalizing, and (**c**) externalizing disorder in victimized children, showing overall agreement between model-predicted risks and observed outcomes. The broken diagonal line represents a perfectly-calibrated model. Error bars represent 95% confidence intervals. Each data-point denotes a probability bin, while its size indicates the relative number of cases within that bin.

17 of 22 predictors were retained in the final LASSO model (see Table 3, **column A**), based on an average *λ* tuning parameter of 0.0109 (range: 0.0075–0.0151). Frequency distributions of predicted risks for victimized children with and without psychopathology at age 18, obtained using nested 10-fold cross-validation, are presented in Fig. 1a. This showed that, beginning at a predicted risk of 0.70, there was a higher proportion of victimized children with versus without any psychiatric disorder in each successive risk class. The ROC curve following internal validation yielded an AUC of 0.69 (95% CI = 0.64–.73; Fig. 2, solid line), representing a 69% probability that a randomly-selected victimized child with any psychiatric disorder will be classified as such by the model compared to a victimized child without psychopathology. The calibration plot (Fig. 3a) showed high agreement between predicted probabilities and observations when the predicted probability was 0.30 and higher; only two participants, or 0.4% of all cases, fell below this point, which likely explains the poorer calibration at this end of the distribution. Calibration-in-the-large (i.e., intercept; α = 0.02) and slope (β = 0.96) did not significantly differ from expected values of a perfectly-calibrated model (*U* = −0.004, χ^2^(2) = 0.06, *p* = .968). Regarding overall performance, the model explained 13.5% of binomial deviance, while the scaled Brier score indicated that the predictors explained 11% of the mean squared error between predicted probabilities and observed outcomes compared to a non-informative model.

#### 3.2.2 Internalizing disorder

18 predictors were retained here (see Table 3**, column B**), based on a mean *λ* of 0.0089 (range: 0.0057–0.0118). There was a higher proportion of victimized children with versus without internalizing disorder between predicted risk thresholds of 0.50 and 0.89 (see Fig. 1b). The internally-validated model yielded an AUC of 0.66 (95% CI = 0.61–.71; Fig. 2, dotted line). Again, calibration-in-the large (α = =−0.10) and slope values (β = 0.75) did not diverge significantly from a perfectly-calibrated model (*U *= 0.002, χ^2^(2) = 3.08, *p* = .214; see Fig. 3b). Indeed, the model was well-calibrated between predicted probabilities of 25% and 60%, within which 89% of cases fell (median = 0.35; interquartile range = 0.27–0.47). The model explained 11.1% of binomial deviance and, based on the scaled Brier score, accounted for 5.8% of mean squared prediction error over a non-informative model.

#### 3.2.3 Externalizing disorder

LASSO regularization selected 15 predictors (see Table 3, **column C**) from a mean *λ* of 0.0146 (range: 0.0105–0.0178). Based on frequency distributions for predicted risk, there was a higher proportion of victimized children with versus without externalizing disorder from a predicted risk of 0.50 and upwards (see Fig. 1c). Discrimination was denoted by an AUC of 0.73 (95% CI = 0.69–.77; Fig. 2, dashed line), while calibration-in-the-large (α = −0.01) and slope (β = 1) values did not diverge significantly from those of a perfectly-calibrated model (*U* = −0.004, χ^2^(2) = 0.003, *p* = .999; see Fig. 3c). Overall, the model explained 15.4% of the deviance, with the scaled Brier score indicating a 15.3% reduction in mean squared error compared to a non-informative model.

### 3.3 Sensitivity analyses

#### 3.3.1 Regularization penalty

For all three outcomes, model performance statistics (see Supplementary **Table S7**) and corresponding plots (see Supplementary **Figs S3** and **S4**) obtained from nested 10-fold cross-validation using less restrictive ‘elastic net’ regularization resembled those generated using LASSO, implying that predictive ability was not significantly worsened by applying the most parsimonious form of regularization to model coefficients.

#### 3.3.2 Non-independence of twins

Average cross-validated performance statistics for each outcome across 10 random single-twin subsamples (*n *= 304–305) resembled those obtained using the full sample (see Supplementary **Tables S8–S10**), suggesting that the inclusion of non-independent observations did not significantly bias predictive accuracy.

## 4 Discussion

We found that factors known to be associated with psychopathology among victimized children can be statistically modelled to predict individual risk for psychopathology during the transition to adulthood. All three models were well-calibrated, and discrimination, while only adequate (AUC  =  0.66–0.73), was within the range of established risk calculators for medical conditions (Damen et al., 2016; Wang et al., 2018), as well as recently-developed prediction models in psychiatry (Cannon et al., 2016; Fusar-Poli et al., 2017; Hafeman et al., 2017). Moreover, because there is currently no evidence base for evaluating individual risk among victimized children, these data-driven approaches are likely to improve on current practice owing to their objectivity and consistency. Our results also suggest a need to consider prediction of internalizing and externalizing psychopathology separately, given observed variations in predictive performance and model configuration; for example, while all five personality dimensions were retained in the prediction of internalizing disorder, only extraversion contributed to the prediction of externalizing disorder.

Our findings should be interpreted in light of several limitations. First, as the E-Risk Study comprises twins, we cannot be certain that results generalize to singletons. However, the prevalence of victimization in our sample is comparable to recent UK general population estimates (Radford et al., 2013), while rates of psychopathology are generally comparable between twins and singletons (Gjone and Novik, 1995). Sensitivity analyses using one twin from each family also suggested that the presence of twins did not inflate predictive accuracy estimates. Second, as findings were based on a community sample of British twins, they may not generalize to other samples, for example children in foster care. External validation is needed to test the relevance of our findings to different contexts before clinical implementation. Third, unlike conventional regression approaches, our prediction models do not permit interpretation of individual coefficients. Specifically, in applying a penalty to reduce over-fitting to the data, LASSO regularization introduces bias into the regression estimates, such that coefficients are no longer reflective of true population-level associations with the outcome. Moreover, LASSO models perform inbuilt variable selection based around the ability of a risk factor to accurately predict variation in the outcome. In this way, we maximize predictive performance in unseen cases but limit model interpretability, as coefficients cannot be used to infer a causal relationship with the outcome; indeed, many variables that are important for prediction may only exert small effects when evaluated based on statistical significance or effect size (Goldstein et al., 2017; Yarkoni and Westfall, 2017). Instead, these coefficients should be viewed as components of the multifactorial risk profile represented by each model's specific combination of predictors.

Despite these limitations, the paper makes important methodological contributions to promote use of prediction modeling techniques in the context of childhood victimization. Compared to previous research, our prospective design ensured appropriate temporal ordering of effects from birth to early adulthood, the developmental point by which most psychiatric disorders have emerged (Kim-Cohen et al., 2003), while a rich characterization of individual-, family-, and community-level characteristics within our sample allowed for comprehensive multivariate models that incorporated potential determinants of both risk and resilience. Moving beyond the group-based average effects estimated in conventional regression analyses, our prediction modeling approach yielded an individualized risk estimate for each victimized child, quantifying their unique likelihood of developing psychopathology. Moreover, our internal validation procedure (i.e., nested cross-validation) ensured that each participant's risk score was calculated when they were excluded from model development, thus reducing over-fitting and increasing the generalizability of our models to unseen cases (Steyerberg, 2009). Finally, automatic variable selection by LASSO regularization suggested that predictors with established univariate-significant associations may not offer an independent contribution to predictive accuracy when considered in a more systemic context.

Overall, these initial findings provide proof-of-principle evidence that a range of individual, family, and community factors can be combined to derive individualized risk scores for psychopathology among victimized young people. With sufficient external validation, prediction modeling has the potential to enhance evidence-based clinical decision-making for this vulnerable population in social work and child and adolescent psychiatry settings and, in turn, inform more rational allocation of limited resources. However, although such risk calculators could introduce greater objectivity and consistency to clinical practice, they should be used to support, rather than replace, professional judgments. Crucially, good predictive performance, even in external samples, does not guarantee clinical usefulness. For example, evaluating overall predictive accuracy across a range of decision thresholds for absolute risk classification (i.e., ‘low-risk’ versus ‘high-risk’), we found that cut-points of 60%, 40%, and 40% provided an optimal balance of sensitivity and specificity in predicting any psychiatric disorder, internalizing disorder, and externalizing disorder, respectively (see Supplementary **Tables S4–S6**). However, the appropriate risk threshold is likely to vary depending on the cost-benefit ratio of the corresponding clinical decision. For example, clinicians could opt for a lower cut-off during initial screening, where the benefit of early detection of true-positive cases may outweigh the cost of higher false-positives, but may prefer a higher threshold for decisions around treatment allocation to ensure that places are allocated to those with the greatest need. Furthermore, classification is a forced choice, and individual predicted probabilities of risk should be presented to both clinicians and patients for an informed choice when all information is available. A decision should not simply rely on a prediction model based only on data included in the prediction model. Therefore, the clinical context, net-benefit and cost-effectiveness of any risk calculator should be evaluated in consultation with key stakeholders (Steyerberg and Vergouwe, 2014).

## 5 Conclusion

The present study provides initial evidence in support of the use of multivariable prediction modeling to derive individualized risk estimates for psychopathology among victimized children. Although we acknowledge a need for external validation and evaluation of clinical usefulness before these models can be confidently integrated into decision-making, they nevertheless offer the potential to supplement current practice, ultimately providing more personalized care for some of the most vulnerable children in society.

## CRediT authorship contribution statement

**Alan J. Meehan:** Formal analysis, Investigation, Writing - original draft. **Rachel M. Latham:** Writing - review & editing. **Louise Arseneault:** Data curation, Funding acquisition, Writing - review & editing. **Daniel Stahl:** Methodology, Writing - review & editing. **Helen L. Fisher:** Conceptualization, Funding acquisition, Writing - review & editing. **Andrea Danese:** Conceptualization, Funding acquisition, Writing - original draft, Writing - review & editing.

## Declaration of Competing Interest

The authors have no conflicts of interest to declare.

## Appendix Supplementary materials

Image, application 1

Image, application 2
