# Supplementary material for: Developing an individualized risk calculator for psychopathology among young people victimized during childhood: A population-representative cohort study
Source: J Affect Disord. 2020 Feb 1;262:90–8. doi: 10.1016/j.jad.2019.10.034 (PMC6916410; doi:10.1016/j.jad.2019.10.034)
Supplement: Supplementary file 2 [file mmc2.docx]

**Supplementary Material for Meehan et al.**

**Developing an individualized risk calculator for psychopathology among young people victimized during childhood: A population-representative cohort study**

**Supplementary Methods**

**The Environmental Risk (E-Risk) Longitudinal Twin Study**

Participants were members of the Environmental Risk (E-Risk) Longitudinal Twin Study, which tracks the development of a nationally-representative birth cohort of 2,232 British twin children. The sample was drawn from a larger birth register of twins born in England and Wales in 1994–1995 (Trouton et al., 2002). Full details about the sample are reported elsewhere (Moffitt and E-Risk Study Team, 2002). Briefly, the E-Risk sample was constructed in 1999-2000, when 1,116 families (93% of those eligible) with same-sex 5-year-old twins participated in home-visit assessments. This sample comprised 56% monozygotic (MZ) and 44% dizygotic (DZ) twin pairs; sex was evenly distributed within zygosity (49% male). Families were recruited to represent the UK population of families with newborns in the 1990s, on the basis of residential location throughout England and Wales and mother’s age. Teenaged mothers with twins were over-selected to replace high-risk families who were selectively lost to the register through non-response. Older mothers having twins via assisted reproduction were under-selected to avoid an excess of well-educated older mothers.

Follow-up home-visits were conducted when children were aged 7, 10, 12 and 18 (participation rates were 98%, 96%, 96% and 93%, respectively). Home-visits at ages 5, 7, 10, and 12 years included assessments with participants as well as their mother (or primary caregiver); the home-visit at age 18 included interviews only with the participants. Each participant in a twin pair was assessed by a different interviewer. There were 2,066 E-Risk participants who were assessed at age 18. The average age of the participants at the time of the assessment was 18.4 years (SD = 0.36); all interviews were conducted after the 18th birthday. There were no differences between those who did and did not take part at age 18 in terms of socioeconomic status (SES) assessed when the cohort was initially defined (χ^2^ = 0.86, *p* = 0.65), age-5 IQ scores (*t* = 0.98, *p* = 0.33), or age-5 internalizing or externalizing behavior problems (*t* = 0.40, *p* = 0.69 and *t* = 0.41, *p* = 0.68, respectively). E-Risk families are representative of UK households across the spectrum of neighborhood-level deprivation: 25.6% of E-Risk families live in “wealthy achiever” neighborhoods compared to 25.3% of households nation-wide; 5.3% vs 11.6% live in “urban prosperity” neighborhoods; 29.6% vs 26.9% live in “comfortably off” neighborhoods; 13.4% vs 13.9% live in “moderate means” neighborhoods; and 26.1% vs 20.7% live in “hard-pressed” neighborhoods (CACI Information Services, 2006; Caspi et al., 2000). E-Risk underrepresents urban prosperity neighborhoods because such households are likely to be childless.

Parents gave informed consent and twins gave assent between 5-12 years and then informed consent at age 18. The Joint South London and Maudsley and the Institute of Psychiatry Research Ethics Committee approved each phase of the study.

**Childhood Victimization**

Exposure to several types of victimization was assessed repeatedly when the children were 5, 7, 10, and 12 years of age, and dossiers have been compiled for each child with cumulative information about: exposure to domestic violence between the mother and her partner; frequent bullying by peers; physical abuse by an adult; sexual abuse; physical neglect; and emotional abuse/neglect. The E-Risk team has previously reported evidence on the reliability and validity of the measures of domestic violence (Moffitt et al., 1997), bullying (Arseneault et al., 2006; Shakoor et al., 2011), physical and sexual abuse (Jaffee et al., 2007; Jaffee et al., 2004), emotional abuse/neglect (Danese et al., 2017), and physical neglect (Fisher et al., 2015). All the component measures are outlined briefly below.

**Physical domestic violence.** Mothers reported about perpetration of and victimization involving 12 forms of physical violence (e.g., slapping, hitting, kicking, strangling) from the Conflict Tactics Scale (CTS; Straus, 1990), on three assessment occasions during the child’s first decade of life (when children were 5, 7, and 10 years of age). Reports of either perpetration or victimization constituted evidence of physical domestic violence. The CTS has between-partner inter-rater reliabilities of 0.76 for perpetration and 0.82 for victimization (Magdol et al., 1998). Families in which no physical violence took place were coded as 0 (55.2%); families in which physical violence took place on one occasion were coded as 1 (28.0%); and families in which physical violence took place on multiple occasions were coded as 2 (16.8%).

**Bullying by peers.** Experiences of victimization by bullies were assessed using both mothers’ and children’s reports. During the interview, the following standard definition of bullying was read out: “Someone is being bullied when another child (a) says mean and hurtful things, makes fun, or calls a person mean and hurtful names; (b) completely ignores or excludes someone from their group of friends or leaves them out on purpose; (c) hits, kicks, or shoves a person, or locks them in a room; (d) tells lies or spreads rumors about them; and (e) other hurtful things like these. We call it bullying when these things happen often, and when it is difficult to make it stop. We do not call it bullying when it is done in a friendly or playful way.” Mothers were interviewed when children were 7, 10, and 12 years old and asked whether either twin had been bullied by another child, responding ‘never’, ‘yes’, or ‘frequently’. We combined mothers’ reports at child age 7 and 10 to derive a measure of victimization during primary school. Mothers’ reports when children were 12 years old indexed victimization during secondary school. During private interviews at 12 years of age, the children indicated whether they had been bullied by another child during primary or secondary school. When a mother or a child reported victimization, the interviewer asked them to describe what happened. Notes taken by the interviewers were later checked by an independent rater to verify that the events reported could be classified as instances of bullying, operationally defined as evidence of (a) repeated harmful actions, (b) between children, and (c) where there is a power differential between the bully and the victim (Shakoor et al., 2011). Although inter-rater reliability between mothers and children was only modest (kappa = 0.20–0.29), reports of victimization from both informants were similarly associated with children’s emotional and behavioral problems, suggesting that each informant provides a unique but meaningful perspective on bullying involvement (Shakoor et al., 2011). We thus combined mother and child reports of victimization to capture all instances of bullying victimization for primary and secondary school separately: reported as not victimized by both mother and child; reported by either mother or child as being occasionally victimized; and reported as being occasionally victimized by both informants or as frequently victimized by either mother or child or both (Bowes et al., 2013). We then combined primary and secondary school ratings to create a bullying victimization variable for the entire childhood period (5–12 years). Children who were never bullied in primary or secondary school, or occasionally bullied during one of these time periods, were coded as 0 (55.5%); children who were occasionally bullied during primary and secondary school, or frequently bullied during one of these time periods, were coded as 1 (35.6%); and children who were frequently bullied at both primary and secondary school were coded as 2 (8.9%).

**Physical and sexual harm by an adult.** We assessed childhood physical and sexual harm in the E-Risk Study using an approach that resembles the process undertaken by child protection agencies. Essentially this is a two-stage process. In child protection, professionals such as teachers working with children typically raise concerns if they observe signs or symptoms or if they become aware of risk that children are victims of violence. When concerns are raised, child protection officers then review the concerns and evaluate them in the context of information previously gathered on that child or family, in order to determine the likelihood that abuse has taken place. In the E-Risk Study, research workers visited the home in pairs, and were extensively trained to detect signs of abuse or neglect. Each time the two research workers visited a home, they interviewed the mother using a structured interview about child harm, tested the children, and observed the family environment using the Home Observation for Measurement of the Environment (HOME; Bradley and Caldwell, 1977). If either research worker had any concerns, they flagged up the case for review. Immediately after each home visit, a review was performed if a family was flagged. In addition, at each wave, any family who had been flagged on a prior wave of the study was automatically reviewed again. Reviews were performed independently by at least two clinical psychologists or psychiatrists and were based on comprehensive dossiers compiled across multiple home visits for each Study member during the course of the ongoing longitudinal study.

An unusual feature of the E-Risk Study’s assessment is that we repeatedly interviewed mothers on four occasions over the years, which allowed them to build confidence in the research team. Also, we were able to reassure mothers that if harm to the child was ongoing and had to be reported by us, reporting would be managed through a trusted familiar professional, namely the family’s registered GP. As the children grew older, some mothers, who were initially reluctant to reveal abuse to us, divulged details of severe abuse at a later interview.

At age 5 we used the standardized clinical protocol from the MultiSite Child Development Project (Dodge et al., 1990; Lansford et al., 2002). At ages 7, 10, and 12 this interview was modified to expand its coverage of contexts for child harm. Interviews were designed to enhance mothers’ comfort with reporting valid child maltreatment information, while also meeting researchers’ responsibilities for referral under the UK Children Act. Specifically, mothers were asked whether either of their twins had been intentionally harmed (physically or sexually) by an adult or had contact with welfare agencies. If caregivers endorsed a question, research workers made extensive notes on what had happened, and indicated whether physical and/or psychological harm had occurred. Under the UK Children Act, our responsibility was to secure intervention if maltreatment was current and ongoing. Such intervention on behalf of E-Risk families was carried out with parental cooperation in all but one case. No families left the Study following intervention.

Over the years of data collection, the Study developed a cumulative profile for each child, comprising the caregiver reports, recorded debriefings with research workers who had coded any indication of maltreatment at any of the successive home visits, recorded narratives of the successive caregiver interviews, and information from clinicians whenever the Study team made a child-protection referral. Each time we visited a home, the research workers flagged concerns, and if there was sufficient evidence to code definite harm then we did so. If evidence only met the level of ‘probable harm’, we kept an ‘ongoing concern list’ and if, at a later wave, there was continued evidence of probable harm, or new evidence, the code was upgraded to ‘definite harm’. Profiles were reviewed at the end of the age-12 phase by two clinical psychologists. Inter-rater agreement between the coders was 90% for cases for whom maltreatment was identified (100% for cases of sexual abuse), and discrepantly coded cases were resolved by consensus review. These were coded as: 0 = ‘no physical harm at any age’; 1 = ‘probable physical harm at any age’; and 2 = ‘definite physical harm at any age’. There were 15.0% of children coded as probably being exposed to physical harm and 5.1% as definitely physically harmed by 12 years of age. There were 0.8% of the children coded as probably exposed to sexual abuse, and 0.7% as definitely exposed to sexual abuse by 12 years of age.

**Emotional abuse and neglect** were coded from research workers’ narratives of the home visits at ages 5, 7, 10, and 12. We coded quite severe examples of parental behavior observed. For example, a mother who had schizophrenia screamed and swore at the children throughout the home visit. As another example, a father who was drunk during the home visit repeatedly spoke abusively to the children in front of the research workers. We found that coders could not empirically separate emotional abuse and emotional neglect in a reliable way, and thus such experiences were coded together as emotional abuse/neglect. Inter-rater agreement between coders exceeded 85% for cases with emotional abuse and neglect, and discrepant cases were resolved by consensus review. Children with no evidence of emotional abuse/neglect were coded as 0 (88.2%), those where there was some indication of emotionally inappropriate/potentially abusive or neglectful behavior were coded as 1 (8.7%), and where there was evidence of severe emotional abuse/neglect, the children were coded as 2 (3.1%).

**Physical neglect.** The cumulative observations of the physical state of the home environment documented by the research workers during home visits to the twins at ages 5, 7, 10 and 12 were reviewed by two raters for evidence of physical neglect. This was defined as any sign that the caretaker was not providing a safe, sanitary, or healthy environment for the child. This included the child not having proper clothing or food, as well as grossly unsanitary home environments (however, this did not include a family living in a deprived or crime-ridden neighborhood). Inter-rater agreement between the coders exceeded 85%, and discrepantly-coded cases were resolved by consensus review. Children with no evidence of physical neglect were coded as 0 (90.9%), those for whom there was an indication of minor physical neglect were coded as 1 (7.1%), and where there was evidence of severe physical neglect the children were coded as 2 (2.0%).

**Psychopathology Outcomes at Age 18**

**Internalizing disorders.** Past-year symptoms of four internalizing disorders were also assessed during private interviews. Generalized anxiety disorder (GAD) and major depressive disorder (MDD) diagnoses were based on *DSM-IV* criteria. Post-traumatic stress disorder (PTSD) diagnosis was derived using *DSM-5* criteria. Eating disorder diagnosis was based on a cut-off of two or more symptoms on the five-item SCOFF screening tool, which consistently detects all positive cases of anorexia nervosa and bulimia nervosa in patient samples (Hill et al., 2010; Morgan et al., 1999). Participants who met criteria for one or more of these four diagnoses were classified as having ‘internalizing disorder’ (37.9% [*n*=209] of 552 victimized participants with available data).

**Externalizing disorders*.*** Past-year symptoms of five externalizing disorders were assessed as part of private interviews with participants at age 18. Attention-deficit/hyperactivity disorder (ADHD) diagnosis was based on *DSM-5* criteria (American Psychiatric Association, 2013). Diagnoses of moderate conduct disorder (CD), alcohol dependence, and cannabis dependence were made using *DSM-IV* criteria (American Psychiatric Association, 1994). Tobacco dependence was diagnosed using the Fagerström Test for Nicotine Dependence (Heatherton et al., 1991). Participants meeting criteria for one or more of these diagnoses were classified as having ‘externalizing disorder’ (43.3% [*n*=239] of 552 victimized children with available data).

**Thought disorder.** Occurrence of psychotic symptoms since age 12 was assessed at age 18, using the same interview previously used to assess psychotic symptoms at age 12 (Polanczyk et al., 2010). Seven items pertaining to delusions and hallucinations were assessed, including “Have other people read your thoughts?”, “Have you thought you were being followed or spied on?”, and “Have you heard voices that other people cannot hear?”. Interviewers coded each item as ‘not’, ‘probably’, or ‘definitely’ present, with coding subsequently verified by a senior psychiatrist. 5.2% (*n*=29) of 558 victimized participants with available data reported having experienced at least one definite psychotic symptom and, thus, were classified as having thought disorder.

**Any psychiatric disorder.** Based on the binary classifications generated for each of the three psychopathological domains, an overall binary outcome for ‘any psychiatric disorder’ was created, where a score of 1 denoted the presence of any internalizing, externalizing, or thought disorder, and a score of 0 indicated that all three were absent. 60.4% (*n*=334) of 553 victimized participants with available data satisfied diagnostic criteria for at least one of the ten constituent psychiatric disorders.

**Childhood Predictors**

Protective factors associated with improved functioning following childhood maltreatment, identified by a recent systematic review of the literature (Meng et al., 2018), were mapped to variables measured in the E-Risk Study between child ages 5 and 12 years (see Supplementary **Table S1**). From those available, we selected a maximum of 22 variables (ensuring an ‘events per variable’ [EPV] ratio of 10 for each outcome), taking the practicability of measures for clinicians into consideration and drawing from the individual, family, and community level. In addition to the factors identified by this systematic review, we included sex and childhood measures of various psychopathology symptoms (attention-deficit/hyperactivity disorder, conduct disorder, anxiety, depression, self-harm/suicide, psychotic symptoms), given the high degree of continuity in psychiatric symptoms from childhood through to adulthood (Rutter et al., 2006).

**Individual-level predictors**

***Sex*** was reported by mothers at study baseline (1 = male; 2 = female).

***IQ*** was tested at age 12 using a short version of the Wechsler Intelligence Scale for Children – Revised (WISC–R; Wechsler, 1974), which comprised two subtests (Matrix Reasoning and Information). We prorated Study members’ IQ according to the method recommended by Sattler (2008). IQ scores ranged from 48 to 143 (M = 96.25, SD = 14.98).

***Personality*** was measured using the child version of the Big Five Inventory (BFI; John and Srivastava, 1999). Study interviewers rated each twin at the end of their two-to-three-hour home visits when the child was aged 12. They rated each twin on the presence of 44 personality characteristics along a three-point rating scale (0 = ‘no’; 1 = ‘a little/somewhat’; 2 = ‘yes’). From these responses, separate scores were derived for each of the ‘Big Five’ personality dimensions: openness to experience (5 items; e.g., perceptive and curious; M = 4.31, SD = 2.76); conscientiousness (6 items; e.g., focused and diligent; M = 8.52, SD = 3.23); extraversion (6 items; e.g., gregarious and talkative; M = 8.28, SD = 3.54), agreeableness (5 items; e.g., considerate and trusting; M = 8.94, SD = 1.70), and neuroticism (5 items; e.g., fearful and touchy; M = 2.08, SD = 1.84).

***Attention-deficit/hyperactivity disorder (ADHD) symptoms*** were measured at age 12 using mother and teacher reports on 18 symptoms of inattention and hyperactivity-impulsivity, drawn from the Achenbach family of instruments (Achenbach, 1991a, b), which were derived from *DSM-IV* ADHD criteria and the Rutter Child Scales (Kuntsi et al., 2004). We averaged the mother- and teacher-rated symptom scores (M = 1.47, SD = 2.57).

***Conduct disorder (CD) symptoms*** at age 12 were derived from mother and teacher reports of child behavioral problems using the Achenbach (1991a, b) family of instruments, and additional *DSM-IV* items. 14 of the 15 *DSM-IV* criteria for CD were assessed, covering aggressive and non-aggressive conduct problems, deceitfulness or theft, and rule violations (‘forced sexual activity’ was excluded as it was considered age-inappropriate). Mother- and teacher-rated symptom counts were averaged (M = 0.61, SD = 1.10).

***Anxiety symptoms*** were assessed when children were aged 12 via direct interviews, using the 10-item version of the Multidimensional Anxiety Scale for Children (MASC; March, 1997). Items were summed to indicate severity of anxiety (M = 7.62, SD = 3.04).

***Depression symptoms*** were assessed at age 12 (via private interviews) using the 27-item Children’s Depression Inventory (CDI; Kovacs, 1992). Items were summed to indicate severity of depression (M = 3.11; SD = 5.32).

***Self-harm and suicide attempts*** were captured by asking mothers whether each twin had ever deliberately harmed themselves or attempted suicide in the previous six months, as part of a face-to-face interview when children were aged 12. Mothers who responded positively to this question were asked to provide a description of the event(s). An independent rater, blind to other data, subsequently used the interview notes to verify that the description provided was clearly an act of self-harm (Fisher et al., 2012). Only mothers were asked to report at this age because of ethical considerations. From this, 2.9% (*n* = 62) of Study members had displayed any self-harming or suicidal behaviors. Examples of self-harming behaviors included cutting and biting arms, pulling out clumps of hair, banging head against walls, and attempted suicide by strangulation.

***Psychotic symptoms*** were evaluated in private interviews conducted by mental health trainees or professionals when the child was aged 12. This interview has been described in detail previously (Polanczyk et al., 2010). To summarize, each child was privately interviewed about seven psychotic symptoms pertaining to delusions and hallucinations, with items including “Have other people ever read your thoughts?”, “Have you ever thought you were being followed or spied on?” and “Have you ever heard voices that other people cannot hear?” A conservative approach was taken in designating a child's report as a symptom. First, the interviewer probed responses using standard prompts designed to discriminate between experiences that were plausible (e.g., “I was followed by a man after school”) and potential symptoms (e.g., “I was followed by an angel who guards my spirit”), and wrote down the child's narrative description of the experience. Second, validity of symptoms was verified by a psychiatrist expert in schizophrenia, a psychologist expert in interviewing children, and a child and adolescent psychiatrist. Third, because children were twins, experiences limited to the twin relationship (e.g., “My twin and I often know what each other are thinking”) were coded as ‘not a symptom’. Children were only classified as experiencing psychotic symptoms if they reported at least one definite symptom. At age 12, 5.9% (*n* = 125) of children reported experiencing at least one definite psychotic symptom.

**Family-level predictors**

***Maternal warmth*** was assessed using procedures adapted from the Five Minute Speech Sample method (Magaña et al., 1986). Mothers were asked to speak for five minutes about each of their children when they were aged 5, and again at age 10. These speech samples were audiotaped and coded by two independent raters, blind to all other E-Risk data, who were shown to have good inter-rater reliability (*r* = .90). The warmth expressed by the mother in their interview about the child was assessed by the tone of voice, spontaneity, sympathy, and/or empathy towards the child. Warmth was coded on a six-point scale, from no warmth (0; complete absence of the defined qualities of warmth) to high warmth (5; definite warmth, enthusiasm, interest in, and enjoyment of the child). As scores for maternal warmth at ages 5 and 10 were significantly correlated (*r* = 0.37, *P* < .001), these were summed to create a single ‘age 5-10’ score (M = 6.96, SD = 1.64).

***Sibling warmth*** was measured by asking mothers a series of questions about the quality of their children’s relationship with one another when the children were aged 7 and 10 (Jaffee et al., 2007). Mothers responded on a three-point scale to six questions (e.g., “Do your twins love each other?”, “Do both your twins do nice things for each other?”). Internal consistency was α = 0.77 at age 7, and α = 0.80 at age 10. As age-7 and age-10 scores were highly correlated (*r* = 0.57, *P* < .001), these were summed to create a single ‘age 7-10’ composite score (M = 19.92, SD = 3.35).

***Adult involvement***. The presence of a supportive adult was assessed at age 12, when children were asked questions about whether they had a stable adult figure to rely on for basic needs and support (e.g., “There is an adult who I can tell almost anything to”; “There is an adult who I can go to if I am in trouble”). These were coded on a three-point scale (0 = ‘not true’ to 2 = ‘definitely true’). We derived a total score by summing responses to 13 items (internal consistency [α] = .85; M = 23.78; SD = 3.44). It should be noted that these questions did not ask the child to specify who the adult was, and thus, this could have been someone within or outside of their family.

***Family history of psychopathology*** was assessed when children were aged 12. In private family history interviews, the twins’ mother reported on her own history of DSM disorders, along with that of her biological mother, father, sisters, and brothers, as well as the twins’ biological father (Milne et al., 2008). This was converted to a proportion (0.0–1.0) of family members with a history of psychiatric disorder (*M* = 0.37, *SD* = 0.27).

***Number of biological parents in household.*** The twins’ living arrangements up to age 10 were assessed by asking mothers whether and when the twins’ biological father was living with the family using a life history calendar (LHC), a visual method that facilitates accurate recall of life events, their timing and duration (Caspi et al., 1996). Children were categorized as follows: ‘constantly lived with both biological mother and father’ (52%; *n* = 1,108); ‘biological father absent at some point’ (40.7%; *n* = 868); ‘biological mother absent at some point’ (1.1%; *n* = 24); and ‘never lived with both biological parents’ (6.2%; *n* = 132). Due to the low prevalence of ‘biological mother absent’, this response was combined with that of ‘biological father absent’, and recoded to reflect the number of biological parents in the household: 0 (never lived with both biological parents; *n* = 132), 1 (one biological parent absent at some point; *n* = 892) and 2 (both biological parents consistently present; *n* = 1,108).

***Socioeconomic status (SES)*** was defined at age 5 using a standardized composite of parental income (total household income), education (highest parent qualification) and occupation (highest parent occupation). These three SES indicators were highly correlated (*r* = 0.57–0.67) and loaded significantly onto one latent factor (Trzesniewski et al., 2006). The population-wide distribution of this latent factor was then divided into tertiles (i.e., low-, medium-, high-SES).

**Community-level predictors**

***Neighborhood crime victimization*** was assessed when children were aged 5 by asking mothers whether they or a member of their family had been the victim of a violent crime (e.g., mugging, assault), a burglary, or a theft in the neighborhood. The three items (each coded 0–2) were summed for each mother, with higher scores indicating greater crime victimization (M = 0.92; SD = 1.31).

***Social cohesion*** was assessed using five items when the children were aged 5 by asking mothers whether their neighborhood was close-knit, whether neighbors shared the same values, whether neighbors trusted and got along with each other, and whether neighbors were willing to help each other (Sampson et al., 1997). We derived a total score by summing these five items (internal consistency [α] = .83), such that higher scores indicated greater social cohesion (M = 7.61; SD = 2.74).

***Status among peers.*** As part of a self-completed computer-based questionnaire at age 12, children reported on their perceived status among their similarly-aged peers. Specifically, they were presented with an image of a target, comprising five concentric circles, and asked to imagine that this represented other children about their age (both within and outside school). In the task instructions, the center circle was described as representing peers who ‘are the center of attention and who are the most important – they usually have a lot of friends.’ In contrast, the outermost circle represented peers who ‘get the least attention and are not so important – they probably don’t have enough friends.’ Children were then asked to indicate which of the five circles they felt they were in. Initial responses were reverse-scored, such that they ranged from 0 (outermost circle; lowest peer status) to 4 (center circle; highest peer status; M = 2.87; SD = 0.97). A pilot study was conducted to validate this task among 49 children aged 10–11, and found a correlation of 0.42 (*p* = .003) with the total score for the Children’s Loneliness Questionnaire (Asher and Wheeler, 1985).

**Statistical Analyses**

**Model development.** Using the *glmnet* package in R (Friedman et al., 2010), separate prediction models were estimated for each age-18 psychiatric outcome via regularized logistic regression. Common explanatory approaches (e.g. logistic regression models), which provide an estimate of average (rather than individual) risk often display ‘over-fitting’; that is, they are overly specific to the unique structure and idiosyncrasies of the data on which they were developed, including any noise or error variance. As a result, estimates of model performance are often over-optimistic, and frequently deteriorate once the model is applied to new data. To combat this, regularized regression places an additional constraint or penalty on the error term of the logistic regression equation (i.e., the binomial deviance), reducing the variance in the predicted values of model coefficients in an effort to mitigate over-fitting and promote generalizability to new or future observations. Importantly, the degree of penalty is determined by maximizing the predictive ability of the unseen cases using cross-validation (see below).

For each of our models, we applied the Least Absolute Shrinkage and Selection Operator (LASSO), which adds the sum of absolute coefficient values to the binomial deviance within the regression equation (Tibshirani, 1996). Our decision to utilize the LASSO penalty was informed by the fact that, unlike some other regularization approaches (such as ridge), coefficients can be reduced to exactly zero, allowing for variable selection. By excluding predictors that do not sufficiently contribute to overall predictive ability, LASSO therefore prioritizes parsimonious models. Where a group of predictor variables are highly correlated, LASSO typically retains only one and shrinks the others to zero; thus, retaining a parsimonious model at a potential cost of slightly lower prediction accuracy (Hastie et al., 2009). However, our predictors were not too highly correlated (*r* = -0.40–0.59).

**Internal validation.** Where wholly independent data are not available, internal validation allows for the evaluation of a model’s predictive ability using ‘unseen’ cases within the current sample. We performed nested 10-fold cross-validation to test each model’s internal validity (Hastie et al., 2009). This procedure consists of two nested cross-validation loops (see Supplementary **Fig. S2**). First, in an outer loop, data are randomly partitioned into ten folds of approximately equal size. Nine of these folds (the blue cubes) are used to estimate or ‘train’ the model, while one fold (the red cube) is retained as ‘test data’ to evaluate model performance. Second, in the inner loop, the training data are used to determine the amount of LASSO regularization applied to model coefficients. This is set by the tuning parameter lambda (*λ*), which can be varied between 0 and 1; as *λ* increases towards 1, coefficient estimates are shrunk closer to zero. In another layer of 10-fold cross-validation, 100 different *λ* values are repeatedly assessed, in order to determine the value that yields the greatest reduction in prediction error for a tenth fold (yellow cube). Third, the *λ* tuning parameter selected by this inner ‘tuning’ loop is applied to the independent ‘test’ fold originally retained in the outer loop (i.e., the red cube), to determine how well this optimal model performs among ‘unseen’ observations that were not involved in model development. Finally, this entire process is repeated using a different ‘test’ fold each time, until all of the ten folds defined in the outer loop have been used to test model performance once.

**Assessing model performance.** Predictive accuracy was evaluated using the class probabilities generated for each individual when they were treated as a test case (i.e., independent from model development) during nested 10-fold cross-validation. Specifically, we examined: (i) discrimination; (ii) calibration; and (iii) overall precision.

Discrimination refers to the model’s ability to distinguish different classes (i.e., correctly classify victimized children with and without psychopathology). This was examined visually using the receiver operator characteristic (ROC) curve, which plots the model’s true positive rate (i.e., sensitivity) against its false positive rate (i.e., 1 – specificity) across various cut-offs or thresholds for predicted risk (Steyerberg, 2009). The area under the curve (AUC) quantifies the model’s overall discriminative ability. The AUC illustrates the probability that a randomly-selected event case will receive a higher ranking than a randomly-selected non-event. For example, an AUC of 0.7 indicates that the likelihood of a randomly-selected participant who actually developed psychopathology being classified as such, compared to a randomly-selected individual who did not, is 70%. An AUC of 0.5 therefore suggests that a model performs no better than chance, while an AUC of 1 indicates perfect discrimination. For logistic regression models, the following benchmarks have been proposed for discrimination: <0.7 = poor, 0.7–0.8 = acceptable, 0.8–0.9 = excellent, and 0.9–1.0 = outstanding (Hosmer et al., 2013).

Calibration captures the level of agreement between predictions and observed outcomes. This is assessed visually by plotting predicted probabilities against observed outcomes, where a 45° line indicates perfect calibration. Predictions are systematically higher than actual observed values where the intercept of this plot (‘calibration-in-the large’) is >0, and lower where it is <0. Meanwhile, a calibration slope <1 suggests over-fitting, or that the model is overly sensitive to the specific data being used, and may not maintain its current predictive ability when applied to new data. Conversely, a slope >1 suggests under-fitting. A statistical test of unreliability (*U*), based on a chi-square test with two degrees of freedom, was used to determine whether the intercept and slope of each calibration plot was significantly different from the perfect diagonal (i.e., intercept of 0; slope of 1; Fenlon et al., 2018).

Finally, overall model performance was assessed using the Brier score, which represents the mean squared difference between predicted probabilities and actual binary outcomes, capturing aspects of both discrimination and calibration (Brier, 1950). Where the incidence of the outcome is 50%, a Brier score of 0 indicates a perfect model, while a score of 0.25 reflects a non-informative model. As the Brier score will vary depending on the incidence of the outcome, we scaled Brier scores by their maximum possible scores under a non-informative model, where Brier_scaled_ = 1 – Brier / Brier_max_ (Steyerberg et al., 2010). This scaled Brier score, ranging from 0% (non-informative) to 100% (perfect), has a similar interpretation to that of Pearson’s *R*^2^ statistic for continuous outcomes (Hu et al., 2006). In this way, Brier_scaled_ corresponds to the proportion of the mean squared difference between predicted and observed values associated with a non-informative model that is accounted for by the current model.

**Description of Sensitivity Analyses**

**Regularization penalty.** LASSO regularization, as utilized in our analyses, performs variable selection, particularly where data are highly correlated, to maximize predictive performance in unseen cases using a more parsimonious set of predictors. Accordingly, we sought to assess whether LASSO regularization may have excluded predictors from our models at the expense of predictive accuracy. Within *glmnet*, the tuning parameter alpha (α), ranging from 0–1, determines the relative weight given to one of two penalty terms that can be applied to the prediction error within the regression model (Friedman et al., 2010). The LASSO penalty (i.e., sum of *absolute* coefficients) is denoted by an α of 1, whereas an α of 0 represents the ridge penalty, which adds the sum of *residual* coefficients to the model’s error term. Ridge regularization does not perform variable selection, but can be better-suited to multicollinear data (James et al., 2013). To test whether a model with the potential to retain more predictors compared to LASSO would show better predictive performance, we re-ran the nested 10-fold cross-validation for each model using ‘elastic net’, a hybrid approach that balances both penalty terms (Zou and Hastie, 2005). Specifically, we set an α of 0.5 as a compromise between ridge and LASSO penalties that, in turn, de-emphasized the variable selection prioritized by full LASSO regularization (i.e., α = 1).

Unstandardized coefficients and model performance statistics for each internally validated elastic net model are presented in Supplementary **Table S7**, alongside the original statistics from LASSO regularization. ROC curves and calibration plots for these sensitivity models are presented alongside their LASSO equivalents in Supplementary **Figs S3** and **S4**, respectively. The same number of predictors were selected for our ‘psychiatric’ and ‘internalizing disorder’ models based on elastic net, while one additional predictor (childhood anxiety symptoms) was retained in the ‘externalizing disorder’ model. Moreover, discrimination, calibration and precision for all three elastic net models closely resembled our LASSO-regularized results. Therefore, applying a less-restrictive form of regularization did not substantially alter the configuration of our models and, in turn, did not improve predictive performance compared to the parsimonious solutions obtained using LASSO.

**Non-independence of twins.** We sought to examine whether the presence of non-independent observations within our twin sample may have biased our measures of model performance. Specifically, higher levels of similarity in the risk profiles of twins (compared to singletons) may have inflated predictive accuracy if, during internal validation, the model was ‘trained’ on one twin and ‘tested’ on the other. To address these concerns, we re-ran nested 10-fold cross-validation with LASSO regularization in 10 subsamples (*n* = 304–305), each consisting of one twin per twin-pair (where both twins were exposed to childhood victimization, one twin was randomly selected). The results of these sensitivity analyses are presented for each model in Supplementary **Tables S8–S10**. Here, Panel A presents model performance statistics (AUC, calibration-in-the-large, calibration slope, Brier score) for the full victimization-exposed sample, while Panel B describes model performance for each of the 10 single-twin subsamples. For each outcome, the average prediction performance across these subsamples was broadly similar to that observed in the full victimized sample, suggesting that the inclusion of twins did not significantly bias our results.

**Supplementary References**

Achenbach, T.M., 1991a. Manual for the Child Behavior Checklist/4-18 and 1991 Profile. University of Vermont Department of Psychiatry, Burlington, VT.

Achenbach, T.M., 1991b. Manual for the Teacher’s Report Form and 1991 Profile. University of Vermont Department of Psychiatry, Burlington, VT.

American Psychiatric Association, 1994. Diagnostic and Statistical Manual of Mental Disorders: DSM-IV, 4th ed. American Psychiatric Association, Washington, DC.

American Psychiatric Association, 2013. Diagnostic and Statistical Manual of Mental Disorders: DSM-5, 5th ed. American Psychiatric Association, Washington, DC.

Arseneault, L., Walsh, E., Trzesniewski, K., Newcombe, R., Caspi, A., Moffitt, T.E., 2006. Bullying victimization uniquely contributes to adjustment problems in young Children: A nationally representative cohort study. Pediatrics 118, 130-138. <https://doi.org/10.1542/peds.2005-2388>.

Asher, S.R., Wheeler, V.A., 1985. Children's loneliness: A comparison of rejected and neglected peer status. J. Consult. Clin. Psychol. 53, 500-505.

Bowes, L., Maughan, B., Ball, H., Shakoor, S., Ouellet-Morin, I., Caspi, A., Moffitt, T.E., Arseneault, L., 2013. Chronic bullying victimization across school transitions: The role of genetic and environmental influences. Dev. Psychopathol. 25, 333-346. <https://doi.org/10.1017/s0954579412001095>.

Bradley, R.H., Caldwell, B.M., 1977. Home Observation for Measurement of the Environment: A validation study of screening efficiency. Am. J. Ment. Defic. 81, 417-420.

Brier, G.W., 1950. Verification of forecasts expressed in terms of probability. Mon. Weather Rev. 78, 1-3.

CACI Information Services, 2006. ACORN User Guide. CACI, London, UK.

Caspi, A., Moffitt, T.E., Thornton, A., Freedman, D., Amell, J.W., Harrington, H., Smeijers, J., Silva, P.A., 1996. The life history calendar: A research and clinical assessment method for collecting retrospective event-history data. Int. J. Methods Psychiatr. Res. 6, 101-114.

Caspi, A., Taylor, A., Moffitt, T.E., Plomin, R., 2000. Neighborhood deprivation affects children’s mental health: Environmental risks identified in a genetic design. Psychol. Sci. 11, 338-242.

Danese, A., Moffitt, T.E., Arseneault, L., Bleiberg, B.A., Dinardo, P.B., Gandelman, S.B., Houts, R., Ambler, A., Fisher, H.L., Poulton, R., Caspi, A., 2017. The origins of cognitive deficits in victimized children: Implications for neuroscientists and clinicians. Am. J. Psychiatry 174, 349-361. <https://doi.org/10.1176/appi.ajp.2016.16030333>.

Dodge, K.A., Bates, J.E., Pettit, G.S., 1990. Mechanisms in the cycle of violence. Science 250, 1678-1683.

Fenlon, C., O’Grady, L., Doherty, M.L., Dunnion, J., 2018. A discussion of calibration techniques for evaluating binary and categorical predictive models. Prev. Vet. Med. 149, 107-114. <https://doi.org/10.1016/j.prevetmed.2017.11.018>.

Fisher, H.L., Caspi, A., Moffitt, T.E., Wertz, J., Gray, R., Newbury, J., Ambler, A., Zavos, H., Danese, A., Mill, J., Odgers, C.L., Pariante, C., Wong, C.C., Arseneault, L., 2015. Measuring adolescents' exposure to victimization: The Environmental Risk (E-Risk) Longitudinal Twin Study. Dev. Psychopathol. 27, 1399-1416. <https://doi.org/10.1017/s0954579415000838>.

Fisher, H.L., Moffitt, T.E., Houts, R.M., Belsky, D.W., Arseneault, L., Caspi, A., 2012. Bullying victimisation and risk of self harm in early adolescence: Longitudinal cohort study. BMJ 344. <https://doi.org/10.1136/bmj.e2683>.

Friedman, J., Hastie, T., Tibshirani, R., 2010. Regularization paths for generalized linear models via coordinate descent. J. Stat. Softw. 33, 1-22.

Hastie, T., Tibshirani, R., Friedman, J., 2009. The Elements of Statistical Learning: Data Mining, Inference, and Prediction, 2nd ed. Springer, New York, NY.

Heatherton, T.F., Kozlowski, L.T., Frecker, R.C., Fagerström, K.O., 1991. The Fagerström Test for Nicotine Dependence: A revision of the Fagerström Tolerance Questionnaire. Br. J. Addict. 86, 1119-1127.

Hill, L.S., Reid, F., Morgan, J.F., Lacey, J.H., 2010. SCOFF, the development of an eating disorder screening questionnaire. International Journal of Eating Disorders 43, 344-351. <https://doi.org/10.1002/eat.20679>.

Hosmer, D.W., Lemeshow, S., Sturdivant, R.X., 2013. Applied Logistic Regression, 3rd ed. John Wiley & Sons, New York, NY.

Hu, B., Palta, M., Shao, J., 2006. Properties of R(2) statistics for logistic regression. Stat. Med. 25, 1383-1395. <https://doi.org/10.1002/sim.2300>.

Jaffee, S.R., Caspi, A., Moffitt, T.E., Polo-Tomas, M., Taylor, A., 2007. Individual, family, and neighborhood factors distinguish resilient from non-resilient maltreated children: A cumulative stressors model. Child Abuse Negl. 31, 231-253. <https://doi.org/10.1016/j.chiabu.2006.03.011>.

Jaffee, S.R., Caspi, A., Moffitt, T.E., Taylor, A., 2004. Physical maltreatment victim to antisocial child: Evidence of an environmentally mediated process. J. Abnorm. Psychol. 113, 44-55. <https://doi.org/10.1037/0021-843x.113.1.44>.

James, G., Witten, D., Hastie, T., Tibshirani, R., 2013. An Introduction to Statistical Learning. Springer, New York, NY.

John, O.P., Srivastava, S., 1999. The Big-Five trait taxonomy: History, measurement, and theoretical perspectives, in: Pervin, L., John, O. (Eds.), Handbook of Personality: Theory and Research. Guilford Press, New York, NY, pp. 102-138.

Kovacs, M., 1992. Children's Depression Inventory (CDI) Manual. Multi-Health Systems, Toronto, ON.

Kuntsi, J., Eley, T.C., Taylor, A., Hughes, C., Asherson, P., Caspi, A., Moffitt, T.E., 2004. Co-occurrence of ADHD and low IQ has genetic origins. Am. J. Med. Genet. B Neuropsychiatr. Genet. 124b, 41-47. <https://doi.org/10.1002/ajmg.b.20076>.

Lansford, J.E., Dodge, K.A., Pettit, G.S., Bates, J.E., Crozier, J., Kaplow, J., 2002. A 12-year prospective study of the long-term effects of early child physical maltreatment on psychological, behavioral, and academic problems in adolescence. Arch. Pediatr. Adolesc. Med. 156, 824-830. <https://doi.org/10.1001/archpedi.156.8.824>.

Magaña, A.B., Goldstein, J.M., Karno, M., Miklowitz, D.J., Jenkins, J., Falloon, I.R., 1986. A brief method for assessing expressed emotion in relatives of psychiatric patients. Psychiatry Res. 17, 203-212.

Magdol, L., Moffitt, T.E., Caspi, A., Silva, P.A., 1998. Developmental antecedents of partner abuse: A prospective-longitudinal study. J. Abnorm. Psychol. 107, 375-389. <https://doi.org/10.1037/0021-843X.107.3.375>.

March, J.S., 1997. Manual for the Multidimensional Anxiety Scale for Children (MASC). Multi-Health Systems, Toronto, ON.

Meng, X., Fleury, M.-J., Xiang, Y.-T., Li, M., D’Arcy, C., 2018. Resilience and protective factors among people with a history of child maltreatment: A systematic review. Soc. Psychiatry Psychiatr. Epidemiol. 53, 453-475. <https://doi.org/10.1007/s00127-018-1485-2>.

Milne, B.J., Moffitt, T.E., Crump, R., Poulton, R., Rutter, M., Sears, M.R., Taylor, A., Caspi, A., 2008. How should we construct psychiatric family history scores? A comparison of alternative approaches from the Dunedin Family Health History Study. Psychol. Med. 38, 1793-1802. <https://doi.org/10.1017/s0033291708003115>.

Moffitt, T.E., Caspi, A., Krueger, R.F., Lynn, M., Gayla, M., Phil, A.S., Ros, S., 1997. Do partners agree about abuse in their relationship? A psychometric evaluation of interpartner agreement. Psychol. Assess. 9, 47-56.

Moffitt, T.E., E-Risk Study Team, 2002. Teen-aged mothers in contemporary Britain. J. Child Psychol. Psychiatry 43, 727-742.

Morgan, J.F., Reid, F., Lacey, J.H., 1999. The SCOFF questionnaire: Assessment of a new screening tool for eating disorders. BMJ 319, 1467-1468.

Polanczyk, G., Moffitt, T.E., Arseneault, L., Cannon, M., Ambler, A., Keefe, R.S.E., Houts, R., Odgers, C.L., Caspi, A., 2010. Etiological and clinical features of childhood psychotic symptoms: Results from a birth cohort. Arch. Gen. Psychiatry 67, 328-338. <https://doi.org/10.1001/archgenpsychiatry.2010.14>.

Rutter, M., Kim-Cohen, J., Maughan, B., 2006. Continuities and discontinuities in psychopathology between childhood and adult life. J. Child Psychol. Psychiatry 47, 276-295. <https://doi.org/10.1111/j.1469-7610.2006.01614.x>.

Sampson, R.J., Raudenbush, S.W., Earls, F., 1997. Neighborhoods and violent crime: A multilevel study of collective efficacy. Science 277, 918-924.

Sattler, J.M., 2008. Assessment of Children: Cognitive Foundations, 5th ed. JM Sattler, San Diego, CA.

Shakoor, S., Jaffee, S.R., Andreou, P., Bowes, L., Ambler, A.P., Caspi, A., Moffitt, T.E., Arseneault, L., 2011. Mothers and children as informants of bullying victimization: Results from an epidemiological cohort of children. J. Abnorm. Child Psychol. 39, 379-387. <https://doi.org/10.1007/s10802-010-9463-5>.

Steyerberg, E.W., 2009. Clinical prediction models: A practical approach to development, validation, and updating. Springer, New York, NY.

Steyerberg, E.W., Vickers, A.J., Cook, N.R., Gerds, T., Gonen, M., Obuchowski, N., Pencina, M.J., Kattan, M.W., 2010. Assessing the performance of prediction models: A framework for some traditional and novel measures. Epidemiology 21, 128-138. <https://doi.org/10.1097/EDE.0b013e3181c30fb2>.

Straus, M.A., 1990. The Conflict Tactics Scales and its critics: An evaluation and new data on validity and reliability, in: Straus, M.A., Gelles, R.J. (Eds.), Physical Violence in American Families: Risk Factors and Adaptations to Violence in 8,145 Families. Transaction Publications, New Brunswick, NJ, pp. 49-73.

Tibshirani, R., 1996. Regression shrinkage and selection via the Lasso. J. R. Stat. Soc. Series B Stat. Methodol. 58, 267-288.

Trouton, A., Spinath, F.M., Plomin, R., 2002. Twins Early Development Study (TEDS): A multivariate, longitudinal genetic investigation of language, cognition and behavior problems in childhood. Twin Res. Hum. Genet. 5, 444-448. <https://doi.org/10.1375/twin.5.5.444>.

Trzesniewski, K.H., Moffitt, T.E., Caspi, A., Taylor, A., Maughan, B., 2006. Revisiting the association between reading achievement and antisocial behavior: New evidence of an environmental explanation from a twin study. Child Dev. 77, 72-88. <https://doi.org/10.1111/j.1467-8624.2006.00857.x>.

Wechsler, D., 1974. Manual for the Wechsler Intelligence Scale for Children—Revised. Psychological Corporation, New York, NY.

Zou, H., Hastie, T., 2005. Regularization and variable selection via the elastic net. J. R. Stat. Soc. Series B Stat. Methodol. 67, 301-320. <https://doi.org/10.1111/j.1467-9868.2005.00503.x>.

**Table S1**

Individual, family, and community resilience factors identified by Meng et al. (2018), mapped to variables measured in the E-Risk Study

| Protective factors identified by systematic review ^a^ | Variables available in E-Risk between ages 5–12 |
| --- | --- |
| **Individual** |  |
| Intelligence | IQ |
| Emotional resources (comprised of: intelligence, positive caregiving, good schools, parental expectations self-esteem, talent, faith, family connectedness, financial resources) | IQ  Socioeconomic status  Maternal warmth |
| Personality | Openness to experience, conscientiousness, extraversion, agreeableness, neuroticism |
| Stable living situation | Number of biological parents in household |
| **Family** |  |
| Socioeconomic status / caregiver education | Socioeconomic status |
| Early family environment / positive family experience / positive or sensitive parenting | Maternal warmth |
| Maternal / parental warmth | Maternal warmth |
| Sibling relationships | Sibling warmth |
| Parental care / years living with biological mother | Number of biological parents in household |
| Maternal depression | Family history of psychopathology |
| Mentorship | Adult involvement |
| Teachers’ & others’ caring | Adult involvement |
| Emotional support | Adult involvement |
| **Community** |  |
| Social support | Adult involvement |
| Living in neighborhood with few problems | Neighborhood crime victimization  Social cohesion |
| Peer relationships | Status among peers |

*Notes*. E-Risk = Environmental Risk Longitudinal Twin Study; IQ = intelligence quotient.

1. See Table 3 in Meng et al. (2018) for summary of resilience/protective factors.

**Table S2**

Descriptive statistics for predictors among severely victimized participants

|  |  | Victimized Sample  (*N* = 555–591) | |
| --- | --- | --- | --- |
| Predictor |  | M | *SD* |
| **Individual** |  |  |  |
| IQ |  | 91.69 | 14.80 |
| Openness to experience |  | 4.10 | 2.80 |
| Conscientiousness |  | 7.77 | 3.56 |
| Extraversion |  | 8.41 | 3.39 |
| Agreeableness |  | 8.73 | 1.86 |
| Neuroticism |  | 2.21 | 1.99 |
| Attention-deficit/hyperactivity disorder symptoms |  | 2.35 | 3.20 |
| Conduct disorder symptoms |  | 1.06 | 1.58 |
| Anxiety symptoms |  | 8.25 | 3.25 |
| Depression symptoms |  | 5.01 | 7.62 |
| **Family** |  |  |  |
| Maternal warmth |  | 6.55 | 1.73 |
| Sibling warmth |  | 18.93 | 3.58 |
| Adult involvement |  | 23.32 | 3.97 |
| Family history of psychopathology |  | 0.50 | 0.28 |
| Socioeconomic status |  |  |  |
| **Community** |  |  |  |
| Neighborhood crime victimization |  | 1.22 | 1.53 |
| Social cohesion |  | 6.78 | 3.19 |
| Status among peers |  | 2.75 | 1.13 |
|  |  |  |  |
|  |  |  |  |
| **Individual** |  | *N* | % |
| Female |  | 294 | 49.8 |
| Any self-harm/suicide attempt |  | 38 | 6.6 |
| 1+ definite psychotic symptoms |  | 67 | 11.6 |
| **Family** |  |  |  |
| Biological parents in household |  |  |  |
| Both parents always present |  | 168 | 29.1 |
| One parent absent at some point |  | 352 | 61.0 |
| Both parents always absent |  | 57 | 9.9 |
| Socioeconomic status |  |  |  |
| Low |  | 304 | 51.4 |
| Middle |  | 164 | 27.8 |
| High |  | 123 | 20.8 |

*Notes*. For continuous variables, the mean (M) and standard deviation (*SD*) is described, while for categorical variables, we include the number (*N*) and proportion (%) of victimized children within a given category.

**Table S3**

Regression equations for individual risk prediction from LASSO regularized regression

| **Probability(Outcome)** | **Regression Equation** |
| --- | --- |
| **P(Any psychiatric disorder) =** | 1 / (1 + exp (– (2.186 – 0.291^*^sex – 0.001^*^IQ + 0.038^*^openness to experience – 0.018^*^conscientiousness + 0.107^*^extraversion – 0.166^*^agreeableness + 0.25^*^CD symptoms + 0.029^*^anxiety + 0.002^*^depression + 1.044^*^psychotic symptoms – 0.310^*^biological parents in household – 0.011^*^adult involvement + 0.033^*^maternal warmth – 0.027^*^sibling warmth + 0.323^*^family history of psychopathology – 0.164^*^SES – 0.047^*^status among peers))) |
| **P(Internalizing disorder) =** | 1 / (1 + exp (– (-1.643 + 0.329^*^sex + 0.037^*^openness to experience – 0.018^*^conscientiousness + 0.090^*^extraversion – 0.059^*^agreeableness + 0.088^*^neuroticism + 0.063^*^CD symptoms + 0.057^*^anxiety + 0.005^*^depression – 0.416^*^self-harm/suicide attempts + 1.112^*^psychotic symptoms – 0.106^*^biological parents in household – 0.031^*^adult involvement + 0.101^*^maternal warmth + 0.709^*^family history of psychopathology – 0.194^*^SES – 0.060^*^neighborhood crime victimization – 0.091^*^status among peers))) |
| **P(Externalizing disorder) =** | 1 / (1 + exp (– (2.558 – 0.811^*^sex – 0.008^*^IQ + 0.048^*^extraversion + 0.005^*^ADHD symptoms + 0.267^*^CD symptoms + 0.005^*^depression + 0.551^*^psychotic symptoms – 0.317^*^biological parents in household – 0.019^*^adult involvement – 0.051^*^sibling warmth + 0.038^*^family history of psychopathology – 0.004^*^SES + 0.070^*^neighborhood crime victimization – 0.023^*^social cohesion + 0.067^*^status among peers))) |

*Notes*. ADHD = attention-deficit/hyperactivity disorder; CD = conduct disorder; LASSO = Least Absolute Shrinkage and Selection Operator; SES = socioeconomic status.

**Table S4**

Performance statistics for the prediction of any psychiatric disorder across a range of potential classification thresholds

| **Risk Score Cut-Off** | **Predicted Risk Prevalence** | **PPV** | **NPV** | **Sensitivity** | **Specificity** |
| --- | --- | --- | --- | --- | --- |
| 20% | 99.8% | 61.1 | 100 | 100 | 0.5 |
| 30% | 97.4% | 62.4 | 92.3 | 99.7 | 6.1 |
| 40% | 88.3% | 64.8 | 67.8 | 93.8 | 20.3 |
| 50% | 72.7% | 68.1 | 58.0 | 81.2 | 40.6 |
| 60% | 53.7% | 71.2 | 50.9 | 62.7 | 60.4 |
| 70% | 28.5% | 81.3 | 47.1 | 38.0 | 86.3 |
| 80% | 13.9% | 87.1 | 43.2 | 19.8 | 95.4 |
| 90% | 5.0% | 96.0 | 40.8 | 7.8 | 99.5 |

*Notes*. Risk score cut-offs begin at 20% due to an absence of cases with risk probabilities below this point in our sample. PPV = positive predictive value (i.e., the proportion of cases classified as having any psychiatric disorder who actually had a disorder); NPV = negative predictive value (i.e., the proportion of cases classified as not having any psychiatric disorder who did not have a disorder). Sensitivity, or the true positive rate, represents the proportion of all positive cases (i.e., those with a psychiatric disorder) who were correctly classified as such. Conversely, specificity, or the true negative rate, is the proportion of negative cases (i.e., those without a psychiatric disorder) correctly classified as such. As an example, using the 50% risk cut-off, 68.1% of cases classified as positive had a psychiatric disorder (PPV), while 58% of predicted negative cases did not have any psychiatric disorder (NPV). The model correctly identified 81.2% of those who had a psychiatric disorder at age 18 (sensitivity), and 40.6% of those without a psychiatric disorder (specificity).

| **Risk Score Cut-Off** | **Predicted Risk Prevalence** | **PPV** | **NPV** | **Sensitivity** | **Specificity** |
| --- | --- | --- | --- | --- | --- |
| 10% | 99.8% | 38.6 | 100 | 100 | 0.32 |
| 20% | 91.7% | 40.0 | 78.6 | 95.4 | 10.7 |
| 30% | 67.9% | 45.6 | 76.5 | 80.4 | 40.0 |
| 40% | 38.1% | 53.7 | 70.8 | 53.1 | 71.3 |
| 50% | 21.0% | 58.5 | 66.8 | 32.0 | 85.8 |
| 60% | 10.5% | 54.7 | 63.4 | 15.0 | 92.3 |
| 70% | 5.4% | 66.7 | 63.1 | 9.3 | 97.1 |
| 80% | 1.4% | 42.9 | 61.6 | 1.5 | 98.7 |
| 90% | 0.2% | 0 | 61.4 | 0 | 99.7 |

**Table S5**

Performance statistics for the prediction of internalizing disorder across a range of potential classification thresholds

*Notes*. PPV = positive predictive value (i.e., the proportion of cases classified as having an internalizing disorder who actually had an internalizing disorder); NPV = negative predictive value (i.e., the proportion of cases classified as not having an internalizing disorder who did not have an internalizing disorder). Sensitivity, or the true positive rate, represents the proportion of all positive cases (i.e., those with an internalizing disorder) who were correctly classified as such. Conversely, specificity, or the true negative rate, is the proportion of negative cases (i.e., those without an internalizing disorder) correctly classified as such. As an example, using the 50% risk cut-off, 58.5% of cases classified as positive had an internalizing disorder (PPV), while 66.8% of predicted negative cases did not have an internalizing disorder (NPV). The model correctly identified 32% of those who had an internalizing disorder at age 18 (sensitivity), and 85.8% of those without an internalizing disorder (specificity).

**Table S6**

| **Risk Score Cut-Off** | **Predicted Risk Prevalence** | **PPV** | **NPV** | **Sensitivity** | **Specificity** |
| --- | --- | --- | --- | --- | --- |
| 20% | 91.9% | 45.7 | 87.8 | 97.7 | 12.5 |
| 30% | 69.5% | 52.4 | 78.6 | 84.8 | 42.0 |
| 40% | 50.7% | 58.6 | 73.1 | 69.1 | 63.2 |
| 50% | 32.3% | 68.1 | 69.0 | 51.2 | 81.9 |
| 60% | 19.2% | 72.2 | 64.0 | 32.3 | 90.6 |
| 70% | 10.5% | 77.4 | 61.1 | 18.9 | 95.8 |
| 80% | 4.4% | 77.3 | 58.6 | 7.8 | 98.3 |
| 90% | 1.8% | 88.9 | 57.9 | 3.7 | 99.7 |

Performance statistics for the prediction of externalizing disorder across a range of potential classification thresholds

*Notes*. Risk score cut-offs begin at 20% due to an absence of cases with risk probabilities below this point in our sample. PPV = positive predictive value (i.e., the proportion of cases classified as having an externalizing disorder who actually had an externalizing disorder); NPV = negative predictive value (i.e., the proportion of cases classified as not having an externalizing disorder who did not have an externalizing disorder). Sensitivity, or the true positive rate, represents the proportion of all positive cases (i.e., those with an externalizing disorder) who were correctly classified as such. Conversely, specificity, or the true negative rate, is the proportion of negative cases (i.e., those without an externalizing disorder) correctly classified as such. As an example, using the 50% risk cut-off, 68.1% of cases classified as positive had an externalizing disorder (PPV), while 69% of predicted negative cases did not have an externalizing disorder (NPV). The model correctly identified 51.2% of those who had an externalizing disorder at age 18 (sensitivity), and 81.9% of those without an externalizing disorder (specificity).

**Table S7**

Unstandardized coefficients and model performance statistics following elastic net regularization, compared to LASSO regularization

|  | Any psychiatric disorder | |  | Internalizing disorder | |  | Externalizing disorder | |
| --- | --- | --- | --- | --- | --- | --- | --- | --- |
|  | Elastic Net | LASSO |  | Elastic Net | LASSO |  | Elastic Net | LASSO |
| **Predictors** | *B* | *B* |  | *B* | *B* |  | *B* | *B* |
| Intercept | 2.285 | 2.186 |  | -1.611 | -1.643 |  | 2.516 | 2.558 |
| **Individual** |  |  |  |  |  |  |  |  |
| Sex (female) | -.297 | -.291 |  | .323 | .329 |  | -.779 | -.811 |
| IQ | -.002 | -.001 |  | – | – |  | -.008 | -.008 |
| Openness to experience | .045 | .038 |  | .039 | .037 |  | – | – |
| Conscientiousness | -.023 | -.018 |  | -.019 | -.018 |  | – | – |
| Extraversion | .102 | .107 |  | .085 | .090 |  | .049 | .048 |
| Agreeableness | -.156 | -.166 |  | -.056 | -.059 |  | – | – |
| Neuroticism | – | – |  | .084 | .088 |  | – | – |
| ADHD symptoms | – | – |  | – | – |  | .012 | .005 |
| CD symptoms | .236 | .250 |  | .060 | .063 |  | .245 | .267 |
| Anxiety symptoms | .029 | .029 |  | .057 | .057 |  | .001 | – |
| Depression symptoms | .003 | .002 |  | .006 | .005 |  | .006 | .005 |
| Self-harm/suicide attempts | – | - |  | -.401 | -.416 |  | – | – |
| Psychotic symptoms | 1.003 | 1.044 |  | 1.072 | 1.112 |  | .533 | .551 |
| **Family** |  |  |  |  |  |  |  |  |
| Maternal warmth | .037 | .033 |  | .097 | .101 |  | – | – |
| Sibling warmth | -.029 | -.027 |  | – | – |  | -.052 | -.051 |
| Adult involvement | -.013 | -.011 |  | -.029 | -.031 |  | -.021 | -.019 |
| Family history of psychopathology | .338 | .323 |  | .689 | .709 |  | .069 | .038 |
| Biological parents in household | -.307 | -.310 |  | -.106 | -.106 |  | -.305 | -.317 |
| Socioeconomic status | -.167 | -.164 |  | -.186 | -.194 |  | -.017 | -.004 |
| **Community** |  |  |  |  |  |  |  |  |
| Neighborhood crime victimization | – | – |  | -.059 | -.060 |  | .071 | .070 |
| Social cohesion | – | – |  | – | – |  | -.024 | -.023 |
| Status among peers | -.051 | -.047 |  | -.089 | -.091 |  | .078 | .067 |
| **Model performance** |  |  |  |  |  |  |  |  |
| **Discrimination** |  |  |  |  |  |  |  |  |
| Area under the curve | 0.69 | 0.69 |  | 0.66 | 0.66 |  | 0.73 | 0.73 |
| **Calibration** |  |  |  |  |  |  |  |  |
| Calibration-in-the-large | 0.012 | 0.016 |  | -0.093 | -0.105 |  | 0.003 | -0.005 |
| Calibration slope | 0.975 | 0.964 |  | 0.781 | 0.754 |  | 1.014 | 0.996 |
| **Brier** | 0.212 | 0.212 |  | 0.222 | 0.223 |  | 0.207 | 0.208 |
| **Brier_scaled_** | 10.9% | 11.0% |  | 6.1% | 5.8% |  | 15.3% | 15.3% |

*Notes*. Mean minimum lambda (*λ*) values for the Elastic Net models were: .0184 (any psychiatric disorder); .0165 (internalizing disorder); .0248 (externalizing disorder). Positive coefficient values mean that higher scores are associated with an increased likelihood of being classified with that disorder at age 18. *B =* unstandardized regression coefficient; ADHD = attention-deficit/hyperactivity disorder; CD = conduct disorder. Brier_scaled_ = 1 – Brier / Brier_max_.

**Table S8**

| **Risk prediction performance measure** | | **Panel A** | **Panel B** | | | | | | | | | | |
| --- | --- | --- | --- | --- | --- | --- | --- | --- | --- | --- | --- | --- | --- |
|  |  | **Victimized participants**  (*n*=505) | **Victimized participants, subsamples consisting of one twin per twin pair**  (*n*=305) | | | | | | | | | | |
|  |  |  | 1 | 2 | 3 | 4 | 5 | 6 | 7 | 8 | 9 | 10 | **Average** |
| **Discrimination** | |  |  |  |  |  |  |  |  |  |  |  |  |
|  | Area under the curve | 0.69 | 0.68 | 0.66 | 0.67 | 0.66 | 0.66 | 0.60 | 0.63 | 0.66 | 0.64 | 0.68 | 0.65 |
| **Calibration** | |  |  |  |  |  |  |  |  |  |  |  |  |
|  | Calibration-in-the-large | 0.02 | -0.16 | 0.08 | 0.01 | 0.03 | 0.10 | 0.13 | 0.10 | 0.03 | 0.08 | 0.06 | 0.06 |
|  | Calibration slope | 0.96 | 1.04 | 0.84 | 0.96 | 0.91 | 0.77 | 0.69 | 0.76 | 0.94 | 0.78 | 0.79 | 0.85 |
| **Overall** | |  |  |  |  |  |  |  |  |  |  |  |  |
|  | Brier | 0.21 | 0.22 | 0.22 | 0.22 | 0.22 | 0.22 | 0.23 | 0.23 | 0.22 | 0.23 | 0.22 | 0.22 |

Twin sensitivity analyses for the prediction of any psychiatric disorder

*Notes*. Panel A shows the internally-validated risk prediction performance of the initial regularized regression model for any age-18 psychiatric disorder among the full victimized sample (see **Table 3, Column A**). We were concerned that using twin (clustered) data may have biased our internal validation results, and undertook a sensitivity analysis to test this, shown in Panel B here. Specifically, we tested ten subsamples consisting of only one twin per twin pair (randomly selected in twin pairs where both were victimized). The average prediction performance was similar to the results of the full victimized sample (Panel A), indicating that the inclusion of twins has not biased our results.

**Table S9**

| **Risk prediction performance measure** | | **Panel A** | **Panel B** | | | | | | | | | | |
| --- | --- | --- | --- | --- | --- | --- | --- | --- | --- | --- | --- | --- | --- |
|  |  | **Victimized participants**  (*n*=504) | **Victimized participants, subsamples consisting of one twin per twin pair**  (*n*=304) | | | | | | | | | | |
|  |  |  | 1 | 2 | 3 | 4 | 5 | 6 | 7 | 8 | 9 | 10 | **Average** |
| **Discrimination** | |  |  |  |  |  |  |  |  |  |  |  |  |
|  | Area under the curve | 0.66 | 0.59 | 0.63 | 0.65 | 0.54 | 0.60 | 0.62 | 0.63 | 0.66 | 0.56 | 0.60 | 0.61 |
| **Calibration** | |  |  |  |  |  |  |  |  |  |  |  |  |
|  | Calibration-in-the-large | -0.10 | -0.18 | -0.11 | -0.09 | -0.28 | -0.24 | -0.08 | -0.07 | -0.09 | -0.15 | -0.08 | -0.14 |
|  | Calibration slope | 0.75 | 0.61 | 0.70 | 0.78 | 0.40 | 0.45 | 0.82 | 0.75 | 0.74 | 0.66 | 0.78 | 0.67 |
| **Overall** | |  |  |  |  |  |  |  |  |  |  |  |  |
|  | Brier | 0.22 | 0.23 | 0.23 | 0.22 | 0.24 | 0.24 | 0.23 | 0.23 | 0.23 | 0.23 | 0.23 | 0.23 |

Twin sensitivity analyses for the prediction of internalizing disorder

*Notes*. Panel A shows the internally-validated risk prediction performance of the initial regularized regression model for age-18 internalizing disorder among the full victimized sample (see **Table 3, Column B**). We were concerned that using twin (clustered) data may have biased our internal validation results, and undertook a sensitivity analysis to test this, shown in Panel B here. Specifically, we tested ten subsamples consisting of only one twin per twin pair (randomly selected in twin pairs where both were victimized). The average prediction performance was similar to the results of the full victimized sample (Panel A), indicating that the inclusion of twins has not biased our results.

| **Risk prediction performance measure** | | **Panel A** | **Panel B** | | | | | | | | | | |
| --- | --- | --- | --- | --- | --- | --- | --- | --- | --- | --- | --- | --- | --- |
|  |  | **Victimized participants**  (*n*=505) | **Victimized participants, subsamples consisting of one twin per twin pair**  (*n*=304) | | | | | | | | | | |
|  |  |  | 1 | 2 | 3 | 4 | 5 | 6 | 7 | 8 | 9 | 10 | **Average** |
| **Discrimination** | |  |  |  |  |  |  |  |  |  |  |  |  |
|  | Area under the curve | 0.73 | 0.69 | 0.69 | 0.71 | 0.69 | 0.72 | 0.68 | 0.72 | 0.68 | 0.67 | 0.69 | 0.69 |
| **Calibration** | |  |  |  |  |  |  |  |  |  |  |  |  |
|  | Calibration-in-the-large | -0.01 | -0.05 | 0.00 | 0.00 | -0.06 | -0.02 | -0.08 | 0.00 | -0.06 | -0.04 | -0.09 | -0.04 |
|  | Calibration slope | 1.00 | 0.84 | 0.98 | 0.97 | 0.86 | 0.94 | 0.81 | 0.98 | 0.85 | 0.91 | 0.79 | 0.89 |
| **Overall** | |  |  |  |  |  |  |  |  |  |  |  |  |
|  | Brier | 0.21 | 0.22 | 0.22 | 0.21 | 0.22 | 0.20 | 0.22 | 0.21 | 0.22 | 0.22 | 0.22 | 0.22 |

**Table S10**

Twin sensitivity analyses for the prediction of externalizing disorder

*Notes*. Panel A shows the internally-validated risk prediction performance of the initial regularized regression model for age-18 externalizing disorder among the full victimized sample (see **Table 3, Column C**). We were concerned that using twin (clustered) data may have biased our internal validation results, and undertook a sensitivity analysis to test this, shown in Panel B here. Specifically, we tested ten subsamples consisting of only one twin per twin pair (randomly selected in twin pairs where both were victimized). The average prediction performance was similar to the results of the full victimized sample (Panel A), indicating that the inclusion of twins has not biased our results.


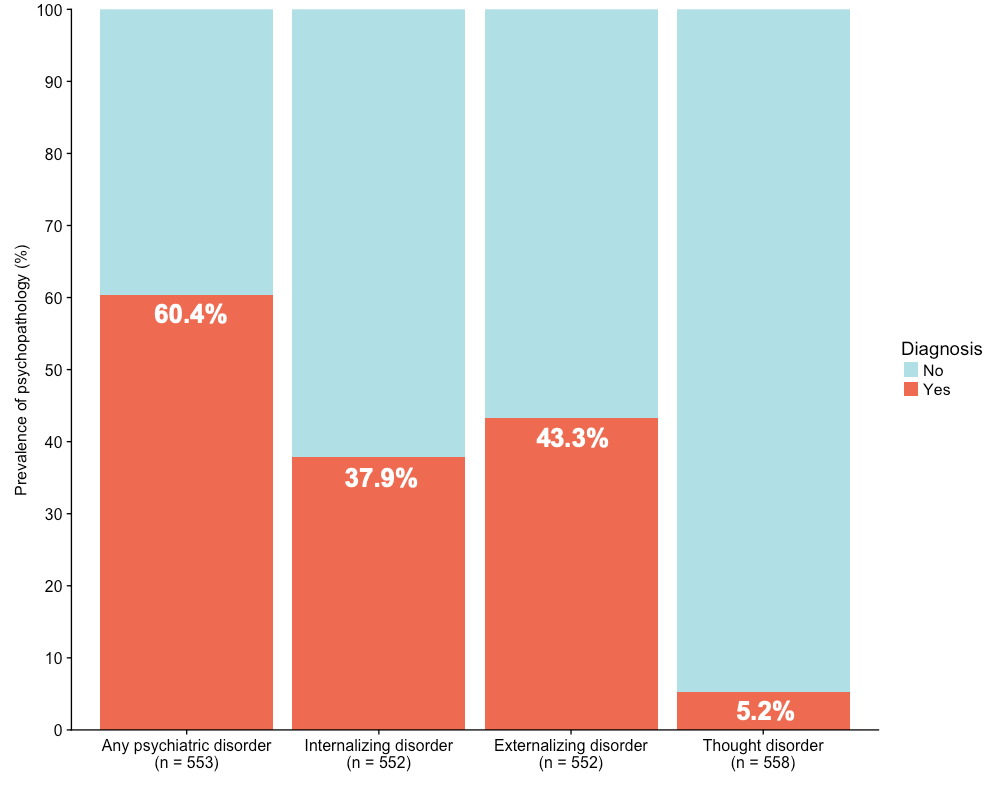


**Fig. S1.** Proportions of victimized children meeting diagnostic criteria for each form of psychopathology.

***
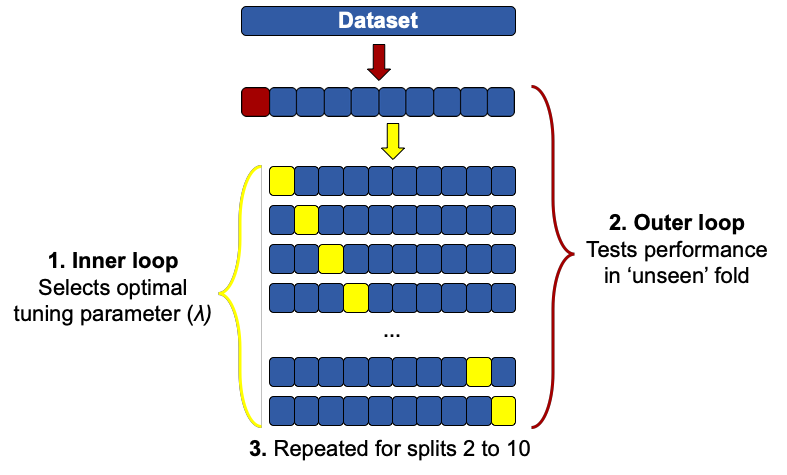
***

**Fig. S2.** Illustrative example of nested 10-fold cross-validation (see Page 14-15 of Supplementary Material for full description).


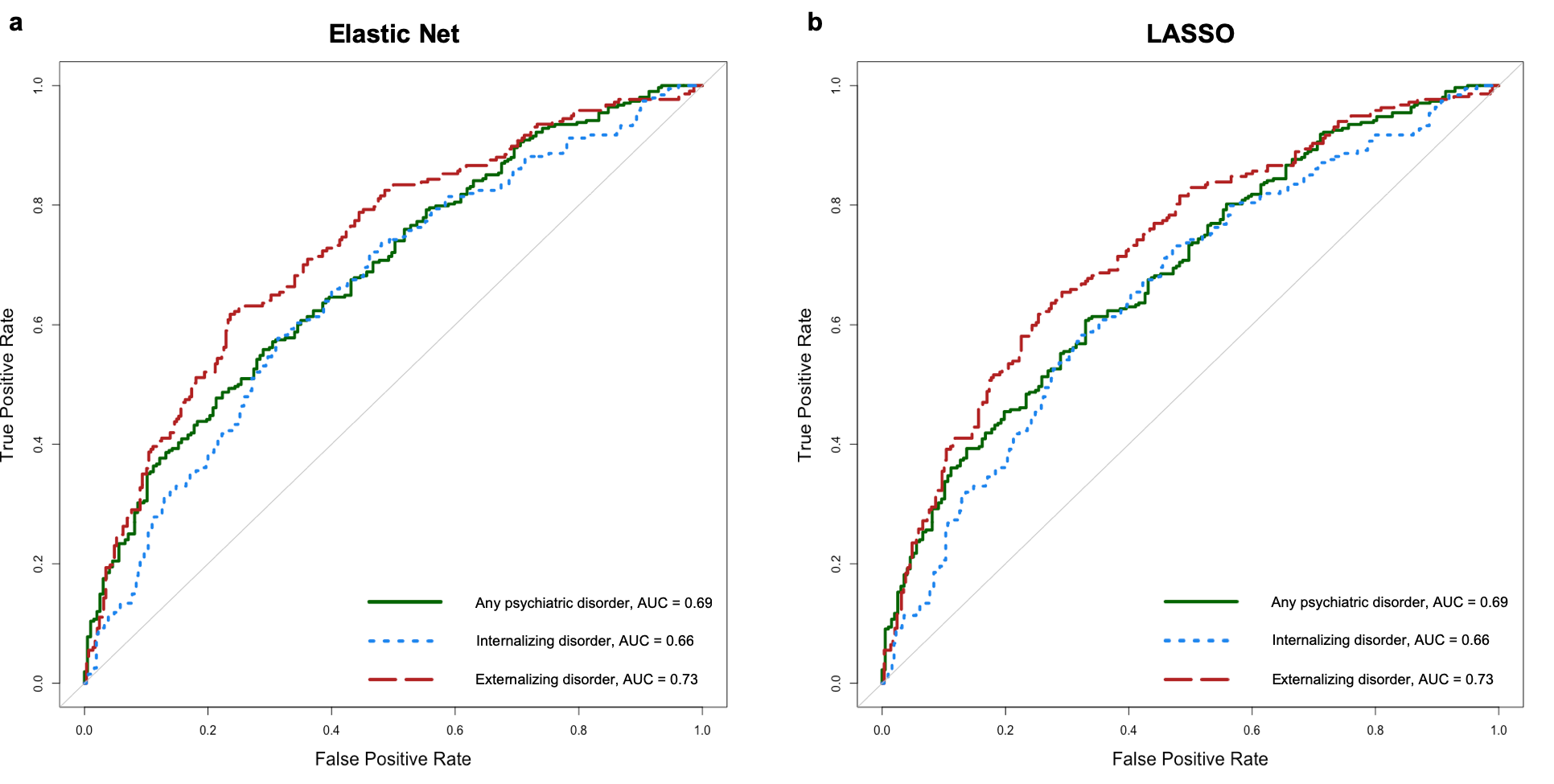


**Fig. S3.** Receiver operating characteristic (ROC) curves for the three psychiatric outcomes at age 18 among victimized children under (**a**) Elastic Net regularization (i.e., α = 0.5); and (**b**) Least Absolute Shrinkage and Selection Operator (LASSO) regularization (i.e., α = 1).


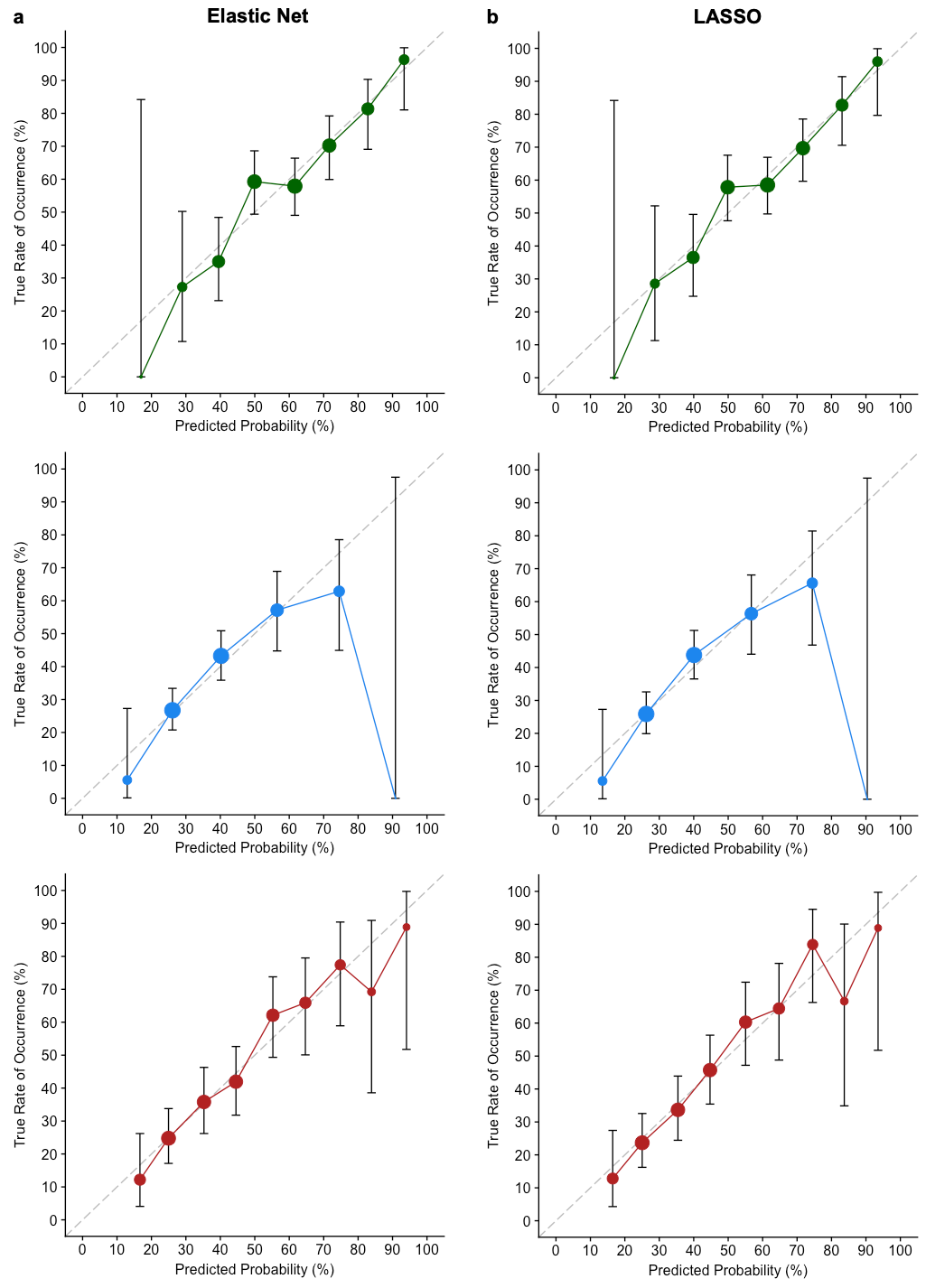
**Fig. S4.** Calibration plots for the three age-18 psychiatric outcomes among victimized children under (**a**) Elastic Net regularization (i.e., α = 0.5); and (**b**) Least Absolute Shrinkage and Selection Operator (LASSO) regularization (i.e., α = 1).
